# Publisher Correction: Random access quantum information processors using multimode circuit quantum electrodynamics

**DOI:** 10.1038/s41467-017-02521-0

**Published:** 2018-01-09

**Authors:** R. K. Naik, N. Leung, S. Chakram, Peter Groszkowski, Y. Lu, N. Earnest, D. C. McKay, Jens Koch, D. I. Schuster

**Affiliations:** 1 The James Franck Institute and Department of Physics, University of Chicago, Chicago, IL 60637 USA; 2 Department of Physics and Astronomy, Northwestern University, Evanston, Illinois 60208 USA; 3 IBM T.J. Watson Research Center, Yorktown Heights, NY 10598 USA

Correction to: *Nature Communications* 10.1038/s41467-017-02046-6, published online 04 December 2017

In the original version of this Article, the affiliation details for Peter Groszkowski and Jens Koch were incorrectly given as ‘Department of Physics, University of Chicago, Chicago, IL, 60637, USA’, instead of the correct ‘Department of Physics and Astronomy, Northwestern University, Evanston, Illinois 60208, USA’. This has now been corrected in both the PDF and HTML versions of the Article.

