# Supplementary material for: Random access quantum information processors using multimode circuit quantum electrodynamics
Source: Nat Commun. 2017 Dec 4;8:1904. doi: 10.1038/s41467-017-02046-6 (PMC5712528; doi:10.1038/s41467-017-02046-6)
Supplement: Supplementary file 1 — Supplementary Information [file 41467_2017_2046_MOESM1_ESM.pdf]

Supplementary Note 1 – Cryogenic setup and control instrumentation

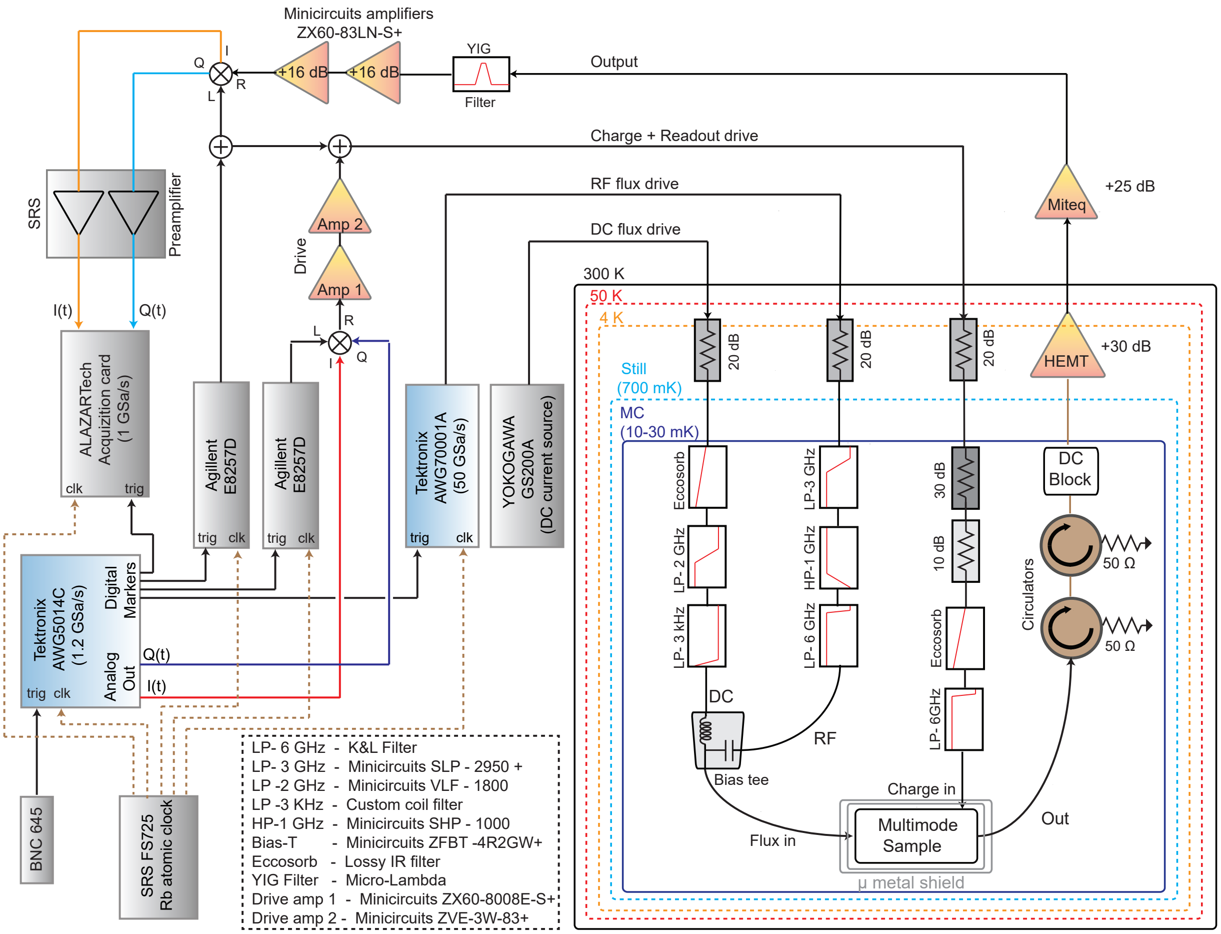

Supplementary Figure 1 | Schematic of the cryogenic setup, control instrumentation, and the wiring of microwave and DC connections to the device.

The device is heat sunk via an OFHC copper post to the base stage of a Bluefors dilution refrigerator (10-30 mK). The sample is surrounded by a can containing two layers of μ-metal shielding, thermally anchored using an inner close fit copper shim sheet, attached to the copper can lid. The schematic of the cryogenic setup, control instrumentation, and the wiring of the device is shown if Supplementary Figure 1. The device is connected to the rest of the setup through three ports: a charge port that applies qubit and readout drive tones, a flux port for shifting the qubit frequency using a DC-flux bias current and for applying RF sideband flux pulses, and an output port for measuring the transmission from the readout resonator. The charge pulses are generated by mixing a local oscillator tone (generated from an Agilent 8257D RF signal generator), with pulses generated by a Tektronix AWG5014C arbitrary waveform generator (TEK) with a sampling rate of 1.2 GSa/s, using an IQ-Mixer (MARQI MLIQ0218). The charge drive pulses are amplified at room temperature (30 dB), and subsequently combined with the readout drive pulse, generated from a second Agilent 8257D RF signal generator, which is also controlled by digital trigger pulses from the TEK. The combined signals are sent to the device, after being attenuated a total of 60 dB in the dilution fridge, using attenuators thermalized to the 4K (20 dB) and base stages (10 + 30 dB). The charge drive line also includes a lossy ECCOSORB CR-117 filter to block IR radiation, and a low-pass filter with a sharp roll-off at 6 GHz, both thermalized to the base stage. The flux-modulation pulses are directly synthesized by a Tektronix AWG70001A arbitrary waveform generator (50 GSa/s) and attenuated by 20 dB at the 4 K stage, and bandpass filtered to within a band of 400 MHz - 3 GHz at the base stage, using the filters indicated in the schematic. The DC flux bias current is generated by a YOKOGAWA GS200 low-noise current source, attenuated by 20 dB at the 4 K stage, and low-pass filtered down to a bandwidth of 3 kHz using a home built C-L-C π filter with a coil inductor (superconducting wire

wound on a spool machined out of Vim Var core iron) and NPO capacitors. The DC flux bias current is combined with the flux-modulation pulses at a bias tee thermalized at the base stage. The state of the transmon is measured using the transmission of the readout resonator, through the dispersive circuit QED readout scheme [1]. The transmitted signal from the readout resonator is passed through a set of cryogenic circulators (thermalized at the base stage) and amplified using a HEMT amplifier (thermalized at the 4 K stage). Once out of the fridge, the signal is filtered (tunable narrow band YIG filter with a bandwidth of 80 MHz) and further amplified. The amplitude and phase of the resonator transmission signal are obtained through a homodyne measurement, with the transmitted signal demodulated using an IQ mixer and a local oscillator at the readout resonator frequency. The homodyne signal is amplified (SRS preamplifier) and recorded using a fast ADC card (ALAZARtech).

Supplementary Note 2 – Device design and fabrication

The CPW resonators in the array have a center pin width of 12 μm and a gap width of 6 μm. They are coupled to each other via interdigitated capacitors, where each side of the capacitor has 6 digits that are 107 μm long, 6 μm wide, and spaced by 6 μm. The capacitor coupling the array to the qubit is identical to the intra-array capacitors to minimize disorder of the resonators in the array. The transmon is capacitively coupled to ground via CPW capacitors on either side of the SQuID, with a center pin width of 20 μm and gap of 10 μm. The SQuID is a 20 μm by 10 μm loop, with two square junctions that are 170 nm and 125 nm wide. The flux bias line 6 μm from the SQuID is dipolar, with 25 μm long and 2 μm wide wires on each end. The ground plane of the chip has an array of 5 μm wide square holes spaced by 50 μm for flux vortex pinning [2]. The linear elements of the full circuit design have been simulated with a commercial 3D finite element analysis software (ANSYS HFSS).

The device (shown in Figure 1 in the main text) was fabricated on a 430 μm thick C-plane sapphire substrate. The base layer of the device, which includes the majority of the circuit (excluding the Josephson junctions of the transmon), consists of 100 nm of aluminum deposited via electron-beam evaporation at 1 Å/s, with features defined via optical lithography and reactive ion etch (RIE) at wafer-scale. The wafer was then diced into 7x7 mm chips. The junction mask was defined via electron-beam lithography with a bi-layer resist (MMA-PMMA) in the Manhattan pattern, with overlap pads for direct galvanic contact to the optically defined capacitors. Before deposition, the overlap regions on the pre-deposited capacitors were milled *in-situ* with an argon ion mill to remove the native oxide. The junctions were then deposited with a three step electron-beam evaporation and oxidation process. First, an initial 35 nm layer of aluminum was deposited at 1 Å/s at an angle of 29° relative to the normal of the substrate, parallel azimuthally to one of the fingers in the Manhattan pattern [3] for each of the junctions. Next, the junctions were exposed to 20 mBar of high-purity O_2 for 12 minutes for the first layer to grow a native oxide. Finally, a second 120 nm layer of aluminum was deposited at 1 Å/s at the same angle relative to the normal of the substrate, but orthogonal azimuthally to the first layer of aluminum. After evaporation, the remaining resist was removed via liftoff in N-Methyl-2-pyrrolidone (NMP) at 80°C for 3 hours, leaving only the junctions directly connected to the base layer, as seen in the inset of Figure 1 of the main text. After both the evaporation and liftoff, the device was exposed to an ion-producing fan for 15 minutes, in order to avoid electrostatic discharge of the junctions.

The device is mounted and wirebonded to a multilayer copper PCB microwave-launcher board. Additional wirebonds connect separated portions of the ground plane to eliminate spurious differential modes. The device chip rests in a pocketed OFHC copper fixture that presses the chip against the launcher board. Notably, the fixture contains an additional air pocket below the chip to alter 3D cavity modes resulting from the chip and enclosure, shifting their resonance frequencies well above the relevant band by reducing the effective dielectric constant of the cavity volume.

Supplementary Note 3 – Multimode quantum memory Hamiltonian

Our multimode quantum memory implementation uses the eigenmodes of a linear array of $n = 11$ identical, strongly coupled superconducting resonators [4], as shown in Figure 1 of the main text. The array is described by the Hamiltonian:

$$\hat{H}_{\rm mm} = \sum_{j=1}^{n} h\nu_{\rm r}\hat{c}_j^\dagger\hat{c}_j + \sum_{j=1}^{n-1} hg_{\rm r}(\hat{c}_j^\dagger\hat{c}_{j+1} + \hat{c}_j\hat{c}_{j+1}^\dagger), \tag{1}$$

where ν_r is the resonance frequency of the identical resonators, g_r is the tunnel-coupling between neighboring resonators, and $\hat{c}_j^\dagger$ ($\hat{c}_j$) is the operator that creates (annihilates) photons in the resonator at spatial index j. The coupling ($g_r \sim 250$ MHz) between neighboring resonators is larger than the disorder in the resonator frequencies ($\nu_r = 6.45\pm0.1$ GHz). Thus, the single-photon eigenmodes of this circuit are 11 distributed "momentum" states of the array, with

eigenfrequencies in a band from 6 to 7 GHz and mode spacing varying from 50 to 150 MHz. The eigenmodes and eigenfrequencies of the tight-binding Hamiltonian of Supplementary Equation (1) are [4]:

$$|\psi\rangle_k = \sum_{j=1}^{n} \sqrt{\frac{2}{n+1}} \sin\left(\frac{jk\pi}{n+1}\right) |1\rangle_j, \tag{2}$$

$$\nu_k = \nu_\mathrm{r} - 2g_\mathrm{r} \cos\left(\frac{k\pi}{n+1}\right) \quad k \in \{1, n\}, \tag{3}$$

where $|\psi\rangle_k$ and ν_k are the kth eigenstate and eigenfrequency, respectively, and $|1\rangle_j$ is the state with a single photon in the jth resonator of the array and with all other resonators in the ground state. The coupling between the transmon and a given eigenmode is given by:

$$g_k = g_\mathrm{q} \sqrt{\frac{2}{n+1}} \sin\left(\frac{k\pi}{n+1}\right). \tag{4}$$

Supplementary Note 4 – Parametric control Hamiltonian

The frequency of the transmon is tunable using the magnetic flux threading the SQuID loop of the transmon, controlled by passing a current through a nearby flux line. For a sinusoidally modulated flux, the transmon $|g\rangle - |e\rangle$ transition frequency is:

$$\nu_\mathrm{ge}(\Phi(t)) = \nu_\mathrm{ge}(\Phi_b + \epsilon_\Phi \sin(2\pi\nu_\mathrm{sb} t + \phi)) \sim \bar{\nu}_\mathrm{ge} + \epsilon \sin(2\pi\nu_\mathrm{sb} t + \phi_\mathrm{m}) \tag{5}$$

where $\bar{\nu}_\mathrm{ge} = \nu_\mathrm{ge}(\Phi_b) + \delta\nu_\mathrm{DC}(\Phi_b, \epsilon_\Phi)$ is the mean qubit frequency during the flux modulation. The relation between the frequency (ϵ) and flux (ϵ_Φ) modulation amplitudes and the DC-shift of the transmon frequency during flux-modulation are:

$$\epsilon = \epsilon_\Phi \left.\frac{d\nu_\mathrm{ge}}{d\Phi}\right|_{\Phi_b} \quad \text{and} \quad \delta\nu_\mathrm{DC} = \frac{\epsilon_\Phi^2}{4} \left.\frac{d^2\nu_\mathrm{ge}}{d\Phi^2}\right|_{\Phi_b}, \tag{6}$$

respectively. The frequency is shifted from its bare value due to the non-linear flux dependence of the transmon frequencies, and is quadratic in the modulation amplitude.

We obtain a simple description for parametric control of this system by considering the Hamiltonian of Equation (1) in the main text and restricting to the lowest two transmon levels. The Hamiltonian then reduces to:

$$\hat{H} = \sum_{k=1}^{n} h\nu_k \hat{b}_k^\dagger \hat{b}_k + \frac{1}{2} h\nu_q(t)\hat{\sigma}_z + \sum_{k=1}^{n} hg_k(\hat{b}_k + \hat{b}_k^\dagger)(\hat{\sigma}_- + \hat{\sigma}_+), \tag{7}$$

The lowest two levels form a qubit, whose frequency is modulated over time by using the flux bias. In this work, we focus on iSWAP interactions between the trasmon and the resonator mode. As a result, we modulate the transmon frequency near the the difference frequencies of the memory modes and transmon. The $(\hat{b}_k\hat{\sigma}_- + \text{c.c.})$ terms in Supplementary Equation (7) can be therefore be dropped in the rotating-wave approximation. When the modulation frequency is resonant with the detuning between the eigenmode and the transmon, this realizes effectively resonant interactions and stimulated vacuum Rabi oscillations of a single photon between the transmon and the mode. These sidebands manifest in a rotating frame defined by the transformation [5, 6]:

$$U(t) = \exp\left[-2\pi i \left(\bar{\nu}_{ge} t - \frac{\epsilon}{2\nu_{sb}} \cos(2\pi\nu_{sb} t)\right)\hat{\sigma}_z - 2\pi i \nu_k \hat{b}_k^\dagger \hat{b}_k t\right]. \tag{8}$$

In this rotating frame, the Hamiltonian is transformed to:

$$\begin{aligned}\hat{H}' = U\hat{H}U^\dagger - iU\partial_t U^\dagger = &\sum_{j=1}^{n} hg_k J_0\left(\frac{\epsilon}{2\nu_\mathrm{sb}}\right)\left(e^{-2\pi i \Delta_k t}\hat{b}_k^\dagger \hat{\sigma}_- + e^{2\pi i \Delta_k t}\hat{b}_k \hat{\sigma}_+\right) \\ &+ \sum_{j=1}^{n} hg_k \hat{b}_k^\dagger \hat{\sigma}_- \left(\sum_{m=1}^{\infty} (-1)^m J_m\left(\frac{\epsilon}{2\nu_\mathrm{sb}}\right) e^{2\pi i (m\nu_\mathrm{sb} - \Delta_k)t}\right) + \text{c.c.},\end{aligned} \tag{9}$$

where $\Delta_k = \nu_k - \bar{\nu}_{ge}$, is the detuning between the qubit and the kth eigenmode. When $\nu_{sb} = \Delta_k$, we obtain resonant first-order sideband transitions between the transmon and mode k, described by:

$$H'_{\text{sb},k} = hg_k J_1\left(\frac{\epsilon}{2\nu_{\text{sb}}}\right)\left(\hat{b}_k^\dagger\hat{\sigma}_- + \hat{b}_k\hat{\sigma}_+\right) \Rightarrow \tau_{\text{iSWAP}} = \frac{1}{2g_k J_1\left(\frac{\epsilon}{2\nu_{\text{sb}}}\right)}. \tag{10}$$

We perform universal operations on the multimode memory using iSWAP operations between a given mode and both the $|g\rangle - |e\rangle$ and $|e\rangle - |f\rangle$ transmon transitions, with the latter allowing the realization of entangling gates between arbitrary eigenmodes. The minimal description of our gate operations on the multimode memory therefore involves three transmon levels, with the parametric control of the eigenmodes described by an extension of the Hamiltonian of Supplementary Equation (9) to a single qutrit coupled to the harmonic memory modes. In addition to sideband transitions, the Hamiltonian also includes dispersive shifts arising from photons in the memory modes, due to the bare coupling between the eigenmodes and the transmon. We ignore the correction to the dispersive shift due to the modulation amplitude dependence of the bare term ($\propto J_0(\epsilon_{\text{m}}/2\nu_{\text{sb}})$), whose lowest order contribution is quadratic in $\epsilon/2\omega_{\text{m}}$. These effects are described by the following simplified Hamiltonian:

$$\tilde{H}\left(t\right) = \tilde{H}_{\text{sb}} + \tilde{H}_{\text{q}} + \tilde{H}_{\chi}, \tag{11}$$

$$\tilde{H}_{\text{sb}} = \sum_k \sum_{\alpha\in ge,ef}\left(g^{\alpha}_{eff,k}(t)\hat{b}_k^\dagger\hat{\sigma}^-_{\alpha}e^{2\pi i(\nu_{sb}-\Delta^{\alpha}_k)t} + \text{c.c.}\right), \tag{12}$$

$$\tilde{H}_{\text{q}} = \sum_{\alpha\in ge,ef}\left(\Omega^{\alpha}\left(t\right)\hat{\sigma}^-_{\alpha} + \text{c.c.}\right), \tag{13}$$

$$\tilde{H}_{\chi} = \sum_k\left(\chi^e_k\left|e\right\rangle\left\langle e\right| + \chi^f_k\left|f\right\rangle\left\langle f\right|\right)\hat{b}_k^\dagger\hat{b}_k. \tag{14}$$

In the above, $\hat{\sigma}^-_{ge} = |g\rangle\langle e|$, $\hat{\sigma}^-_{ef} = |e\rangle\langle f|$ and $g^{\alpha}_{\text{eff},k}(t) = g^{\alpha}_k J_1\left(\frac{\epsilon^{\alpha}_k(t)}{2\nu_{\text{sb}}}\right)$. $\epsilon^{\alpha}_k(t)$ for $\alpha \in \{ge, ef\}$ are the strengths of time-dependent parametric frequency modulation tones addressing mode k and $\Delta^{\alpha}_k = \nu_k - \bar{\nu}_{\alpha}$ is the detuning between mode k and the frequency of the corresponding transmon transition frequency $\alpha \in \{ge, ef\}$. $\Omega^{\alpha}(t)$ are the strengths of the transmon charge drives and $\chi^{e,f}_k$ are the dispersive shifts of the $|e\rangle$ and $|f\rangle$ levels resulting from the addition of a photon in mode k. In addition to the dispersive shift, there are second-order tunneling terms of the form $b_l^\dagger b_k$ for $l \neq k$ arising from the virtual hopping of photons between different eigenmodes via the transmon. These terms are of the same order as the dispersive shift, but their effect can be ignored since they correspond to off-resonant tunneling (~ 1 MHz) between non-degenerate levels (spaced by ~ 100 MHz). We note that there is also a shift (DC-offset) of the qubit frequency during the flux modulation, arising from the non-linear flux-frequency relation of the transmon. Given that the flux pulses used in the experimental sequences in this work are sequential, we include their effect as:

$$\tilde{H}_{\text{DC}} = \sum_{j;\beta\in\{e,f\}}\delta\nu^{j,\beta}_{\text{DC}}\left(\epsilon^{\beta}_j(t)\right)\left|\beta\right\rangle\left\langle\beta\right|, \tag{15}$$

with additional cross terms being present if different flux tones were turned on simultaneously. We can further simplify the Hamiltonian above by ignoring off-resonant terms. If the transmon charge and flux-modulation tones are of the form $\epsilon_{q,\text{sb}}\cos\left(\omega_{q,\text{sb}}t + \phi_{q,\text{sb}}\right)$, and we consider near resonant operations with a single eigenmode k, the drive phases ($\phi_{q,\text{sb}}$) enter the effective Hamiltonian as:

$$\tilde{H}_{\text{sb},\alpha}(t) = g_{\text{sb},\alpha}(t)\hat{a}_k^\dagger\hat{\sigma}^-_{\alpha}e^{-i\phi_{\text{sb}}} + \text{c.c.} \qquad \tilde{H}_{\text{q}} = \Omega_{\alpha}(t)\hat{\sigma}^+_{\alpha}e^{-i\phi_q} + \text{c.c.} \quad \alpha \in \{ge, ef\} \tag{16}$$

Reducing to 2×2 subspaces over which each of these terms act, and taking the top row to be the state with the higher transmon level, and with $\theta(t) = 2\Omega_{\alpha}t$ and $2g_{\text{sb},\alpha}t$ for the sideband and qubit drives, we obtain:

$$\hat{H}_{\text{sb},ge}(t) = \begin{pmatrix} 0 & g_{\text{sb},ge}e^{i\phi_{\text{sb}}} \\ g_{\text{sb},ge}e^{-i\phi_{\text{sb}}} & 0 \end{pmatrix} \Rightarrow \hat{U}^{\theta}_{\text{sb},ge} = \begin{pmatrix} \cos\left(\frac{\theta}{2}\right) & -i\sin\left(\frac{\theta}{2}\right)e^{i\phi_{\text{sb}}} \\ -i\sin\left(\frac{\theta}{2}\right)e^{-i\phi_{\text{sb}}} & \cos\left(\frac{\theta}{2}\right) \end{pmatrix}, \tag{17}$$

$$\hat{H}_{\text{q},ge}(t) = \begin{pmatrix} 0 & \Omega_{ge}e^{-i\phi_{\text{q}}} \\ \Omega_{ge}e^{i\phi_{\text{q}}} & 0 \end{pmatrix} \Rightarrow \hat{U}^{\theta}_{\text{q},ge} = \begin{pmatrix} \cos\left(\frac{\theta}{2}\right) & -i\sin\left(\frac{\theta}{2}\right)e^{-i\phi_{\text{q}}} \\ -i\sin\left(\frac{\theta}{2}\right)e^{i\phi_{\text{q}}} & \cos\left(\frac{\theta}{2}\right) \end{pmatrix}. \tag{18}$$

Supplementary Note 5 – Transmon and readout resonator properties

The parameters of the transmon are obtained by fitting the spectrum obtained as a function of the applied DC flux. The Josephson and electrostatic charging energies extracted from these fits are $E_{\text{J,max}} = 22.2$ GHz and $E_{\text{C}} = 192$

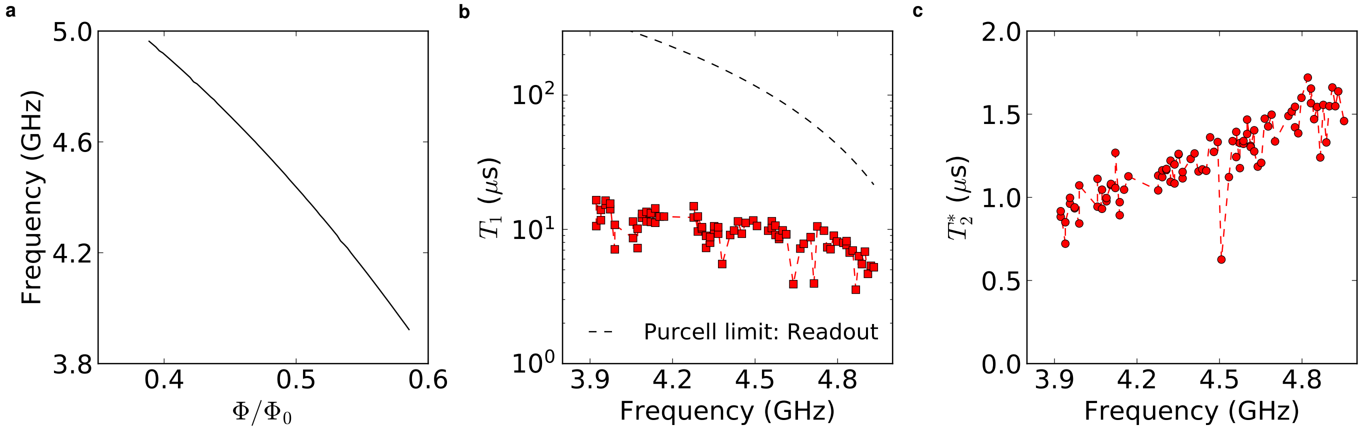

Supplementary Figure 2 | a, Qubit frequency as a function of applied DC flux bias in units of flux quanta. **b,** Energy relaxation time (T_1) as a function of transmon frequency, with the Purcell limit from the readout resonator shown for comparison. **c,** Ramsey (T_2^*) coherence times as a function of transmon frequency.

MHz, while the SQuID loop junction asymmetry, $(E_{\mathrm{J1}} - E_{\mathrm{J2}})/(E_{\mathrm{J1}} + E_{\mathrm{J2}}) = 0.1$. These parameters correspond to maximum and minimum qubit frequencies of 5.84 GHz and ~ 2 GHz, respectively. The experiments in this work were typically performed with the transmon biased between $3.9 - 4.7$ GHz (see Supplementary Figure 2a). This frequency band is ~ 2 GHz away from the eigenmodes of the resonator array. As a result, photons in the multimode manifold cause relatively small dispersive shifts of the transmon frequency. Additionally, the slope of the flux-frequency curve in this regime allows for sufficiently large frequency modulation amplitudes, while limiting sensitivity to flux noise to maintain transmon coherence. The transmon qubit state is probed using a capacitively coupled CPW readout resonator. The frequency and the quality factor of the readout resonator are $\nu_{\mathrm{read}} = 5.255$ GHz and $Q = 15000$, and the coupling to the qubit is $g_{\mathrm{read}} = 47$ MHz. For the typical transmon frequency range, we obtain single-shot readout fidelities between $0.3 - 0.85$ using dispersive [1] and high-power [7] circuit QED readout schemes. The readout signal is calibrated by appending a sequence with no pulse, and one with a transmon $|g\rangle - |e\rangle$ π pulse at the of each set of experimental sequences. Upon averaging over 1000-2000 experiments, the readout signal results in a visibility of $\sim 99\%$, limited by the fidelity of the single qubit gates, and consistent with the RB data.

The coherence of the transmon is characterized by standard lifetime (T_1) and Ramsey (T_2^*) experiments. The measured T_1 of the $|e\rangle$ state and T_2^* of the $|g\rangle - |e\rangle$ transition are show as a function of the $|g\rangle - |e\rangle$ transition frequency in Supplementary Figure 2b and 2c. The T_1 is found to show a slight decrease with increasing frequency in this regime, explained partially by increased Purcell loss from coupling to the readout resonator (see Supplementary Figure 2b). The T_1 at a given flux bias slowly varies with time (over the course of weeks) by $\sim 25 - 30\%$. The T_2^* is found to increase with frequency, consistent with reduced sensitivity to flux noise due to the decreasing slope of the frequency-flux curve transform. We note that T_2^* showed no improvement from reducing the cutoff frequency of the external cryogenic low-pass filter on the DC flux bias from 100 to 3 kHz. At $\nu_{\mathrm{q}} = 4.36$ GHz, the T_2 obtained from a spin-echo experiment with a single π pulse is 3.7 μs. This time could be increased to ~ 14 μs using a dynamical decoupling sequence (Carr-Purcell-Meiboom-Gill with 61 pulses) [8]. We note that the measured T_2^* jumped from 400 ns to 1.2 μs, 2-3 weeks following cooling the fridge to the base temperature (20 mK), coincident with a shift and stabilization of the applied current corresponding to a flux quantum. The coherence of the $|f\rangle$ level is characterized by analogous lifetime and Ramsey experiments. The lifetime of the $|f\rangle$ level at $\nu_{\mathrm{q}}^{ge} = 4.3325$ GHz is $T_{1,ef} = 3.7$ μs while the phase coherence time is $T_{2,ef}^* = 1.2$ μs.

Supplementary Note 6 – Stimulated vacuum Rabi oscillations with multiple transmon levels

The eigenmodes of the resonator array are probed by sideband spectroscopy using the RF flux bias (see Supplementary Figure 1). The flux-modulation pulses are directly synthesized using a Tektronix AWG70000A arbitrary waveform generator (AWG) with a sampling rate of 50 GSa/s. The target pulse waveform envelopes are typically square, with Gaussian edges ($\sigma = 10 - 20$ ns, 2σ cutoff) added to reduce the pulse bandwidth and thus minimize crosstalk between modes. The protocol used to the measure the $|g\rangle - |e\rangle$ sideband spectrum is shown in Figure 2 of the main text and reiterated in Supplementary Figure 3a, with the corresponding measured spectrum shown in

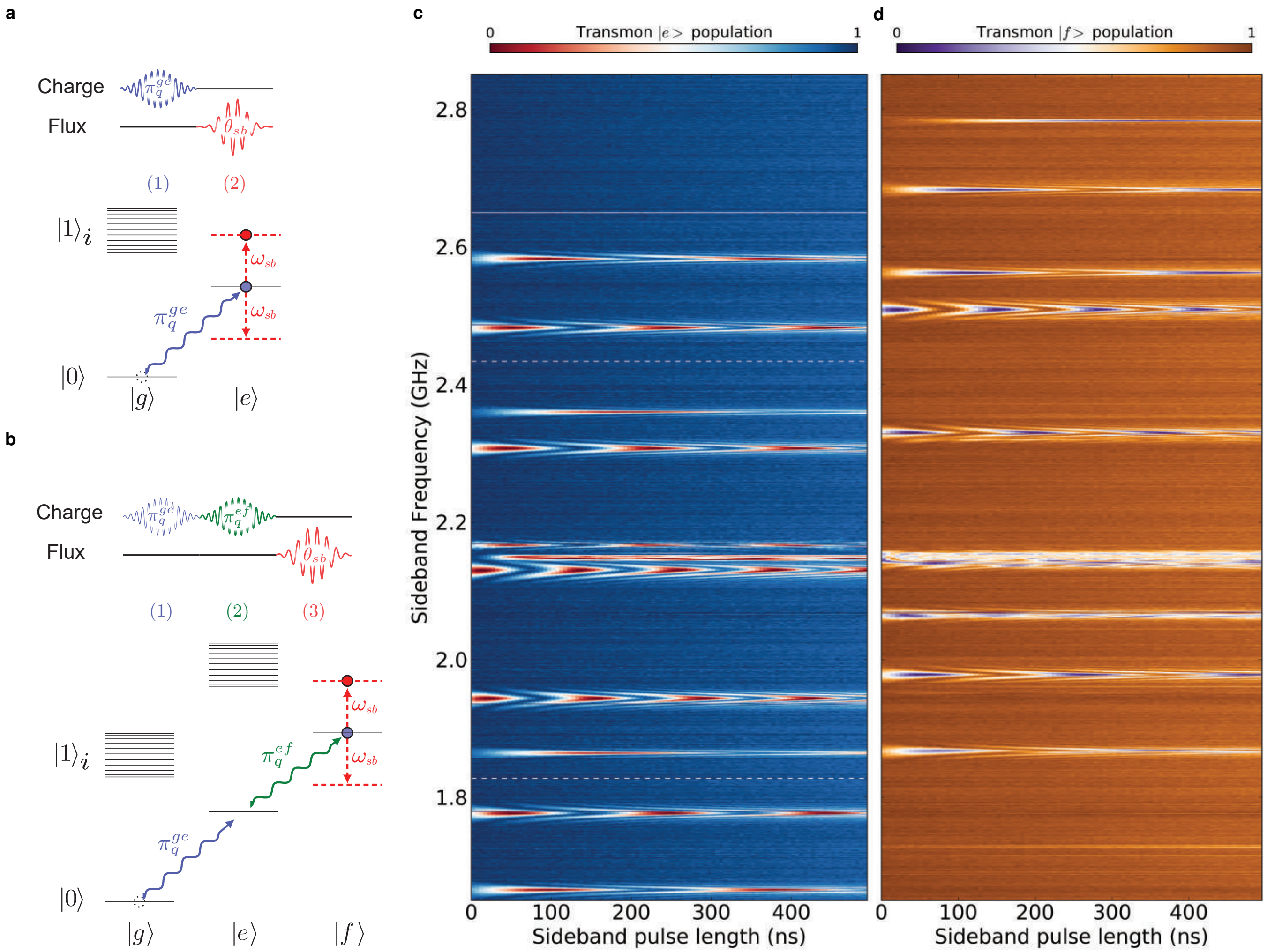

Supplementary Figure 3 | a and b, Level diagrams and experimental protocols for probing sideband transitions across $|g\rangle - |e\rangle$ and $|e\rangle - |f\rangle$, respectively. **c and d,** The corresponding stimulated vaccum Rabi spectra with the transmon biased at $\nu_{ge} = 4.3325$ GHz. These spectra are taken at a fixed flux-modulation amplitude, after correcting the distortion of the flux pulses due to the transmission profile of the flux bias (see Supplementary Note 7). At this drive amplitude, only nine of the 11 modes can be resolved, with the two remaining modes being weakly coupled. The locations of the two weakly coupled modes, found by driving at higher flux-modulation amplitudes, are indicated by the white dashed lines in **c**.

Supplementary Figure 3c . The spectrum for $|e\rangle - |f\rangle$ sideband transitions are obtained using a similar protocol (see Supplementary Figure 3b), beginning instead by first loading the transmon in the $|f\rangle$ state. Following a sideband flux pulse with frequency $\nu_{\rm sb}$ and duration τ, we measure the population in the transmon $|f\rangle$ state. The $|f\rangle$ state population is obtained by mapping $|e\rangle - |f\rangle$ to $|g\rangle - |e\rangle$, and subsequently measuring the $|e\rangle$ population as before. The spectrum thus obtained as a function of $\nu_{\rm sb}$ and τ is plotted in Supplementary Figure 3d. These spectra are taken at a fixed frequency-modulation amplitude, after calibrating and correcting for the transfer function of the RF flux bias as described in the following section.

Supplementary Note 7 – Correcting for the transmission profile of the flux bias line

We directly calibrate the frequency-modulation amplitude using the DC-shift of the qubit frequency during flux modulation. This frequency shift is measured using a transmon Ramsey interferometry experiment, with a flux pulse inserted during the idle time (Supplementary Figure 4b inset). For a fixed external RF voltage amplitude, the measured DC-offset as a function of the flux-modulation frequency is shown in Supplementary Figure 4b, along

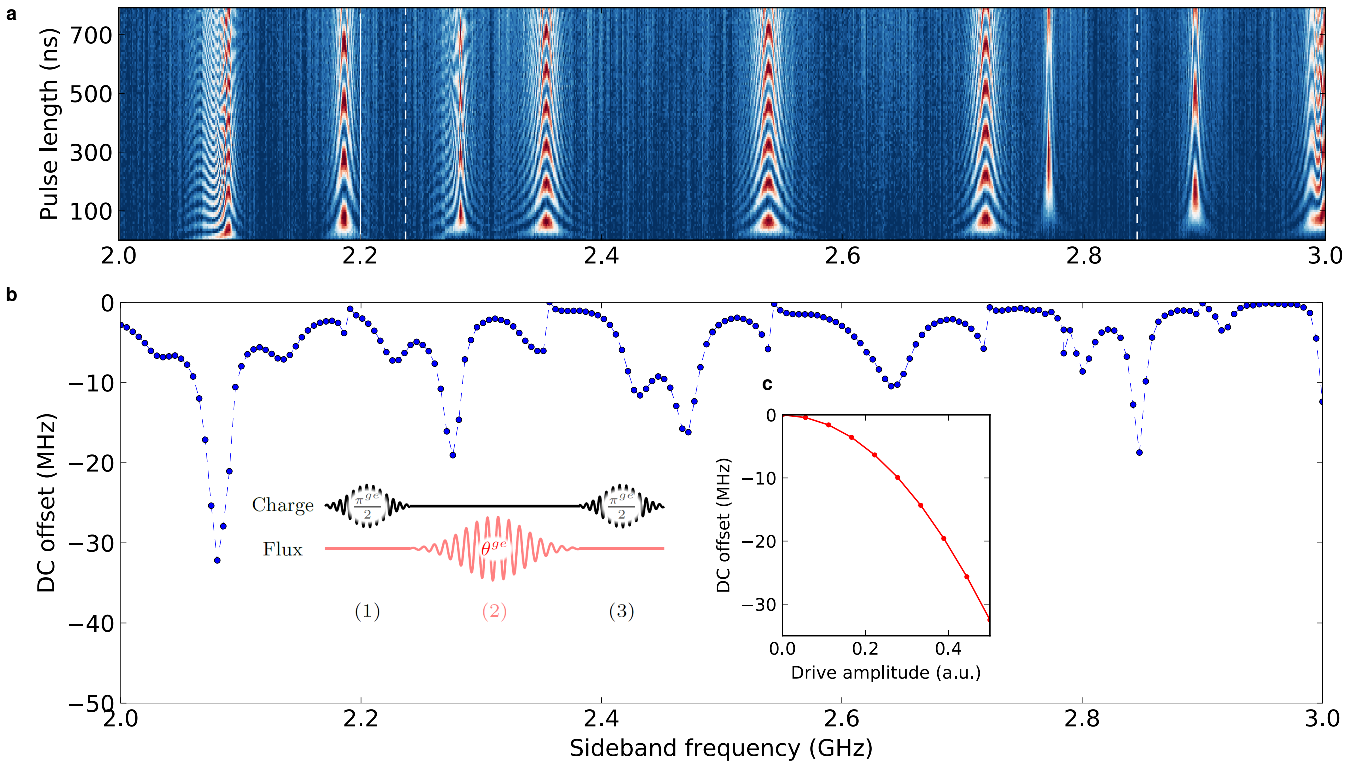

Supplementary Figure 4 | a, Sideband spectrum on the $|g\rangle - |e\rangle$ transition with the transmon biased at $\nu^{ge} = 3.9225$ GHz. **b,** Qubit DC-offset at a fixed drive amplitude as a function of sideband frequency measured using a transmon Ramsey experiment on the $|g\rangle - |e\rangle$ transition with a flux pulse inserted during the idle time. **c,** DC-offset as a function of drive amplitude for $\nu_{\rm sb} = 2.63$ GHz, showing the expected quadratic dependence with drive amplitude.

with the corresponding $|g\rangle - |e\rangle$ sideband spectrum is shown in Supplementary Figure 4a. The stimulated vacuum Rabi chevrons for some modes are found to be distorted from the expected shape [6] (see mode 0 and 10). These distortions are due to resonances in the transmission profile of the flux bias line, as seen in the DC-offset spectroscopy. The memory modes appear as avoided crossings in a Ramsey experiment on $|g\rangle - |e\rangle$ transition, due to interference of Ramsey fringes arising from the DC-offset and resonant stimulated vacuum Rabi oscillations. At a given frequency, the DC-offset shows a quadratic dependence on the modulation amplitude seen in Supplementary Figure 4(c), as expected from equation (6). The amplitude profile of the transfer function $T(\nu)$ of the flux bias line is obtained from the DC-offset at fixed AWG drive voltage, with $|T(\nu)| \propto \sqrt{|\delta\nu_{\rm DC}|}$.

For short pulse durations and large stimulated vacuum Rabi rates, the bandwidth of the pulse becomes commensurate with the frequency scale over which the transfer function of the flux bias varies significantly, causing distortion of the flux pulses. However, this effect is corrected using the knowledge of the transfer function of the flux bias. The complete complex transfer function (characterizing the amplitude and the phase of the flux bias distortion) is extracted only from the amplitude of the transfer function, by assuming that the response of the line is causal and enforcing the Kramers-Kronig relations [9]. We enforce these relations here by fitting the amplitude of the measured transfer function to the functional form:

$$|T(\nu)| = \left| y_0 + \sum_i \frac{A_i}{(\nu^2 - \nu_{0,i}^2) - i\gamma\nu} \right| . \tag{19}$$

We account for flux-pulse distortion by modifying the pulses generated by the AWG to account for the transfer function of the flux bias line. The AWG waveform used to generate a given pulse $f(t) = \mathrm{Re}[f_{\rm c}(t)]$ at the location of the qubit is:

$$f_{\rm AWG}(t) = \mathrm{Re}\left[\mathrm{IFFT}\left(\frac{\mathrm{FFT}(f_{\rm c}(t))}{T(\nu)}\right)\right] . \tag{20}$$

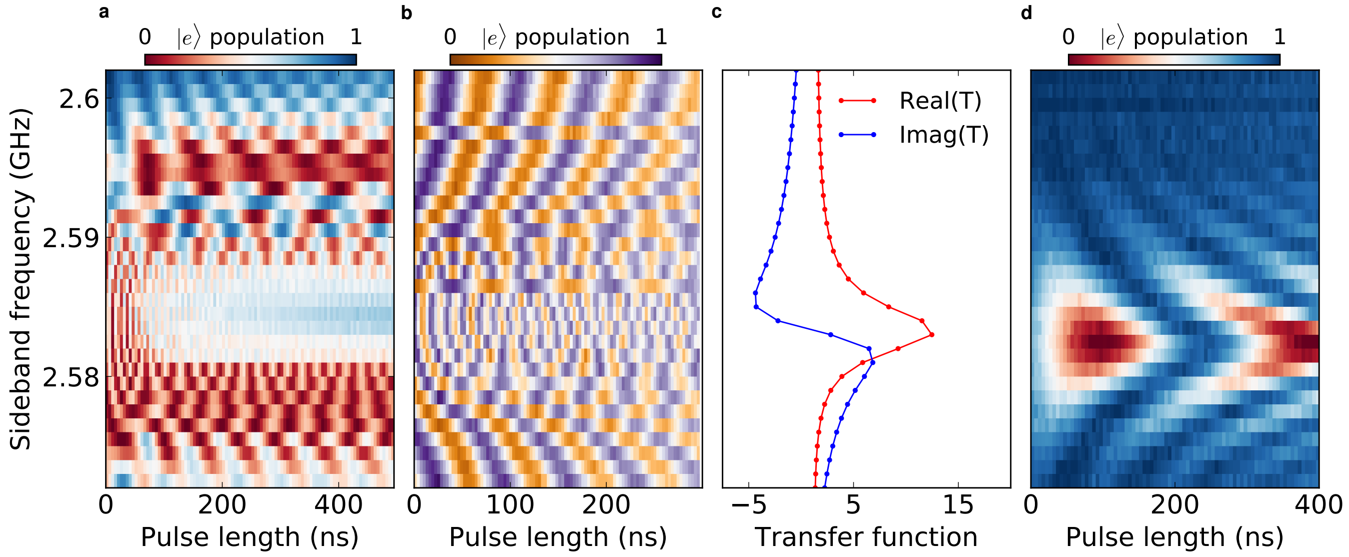

Supplementary Figure 5 | a, Stimulated vacuum Rabi spectrum on the $|g\rangle - |e\rangle$ transition near mode 10 with the transmon biased at 4.3325 GHz. **b,** Ramsey experiment on the $|g\rangle - |f\rangle$ transition with a flux-pulse inserted during the idle time. This allows for a precise measurement of the line attenuation near the modes. **c,** Real and Imaginary parts of the Transfer function obtained by fitting the experimentally measured transfer function amplitude to Supplementary Equation (19). This form is automatically constrained to be causal, with the real and imaginary parts satisfying the Kramers-Kronig relations. **d,** Corrected spectrum, revealing a chevron pattern.

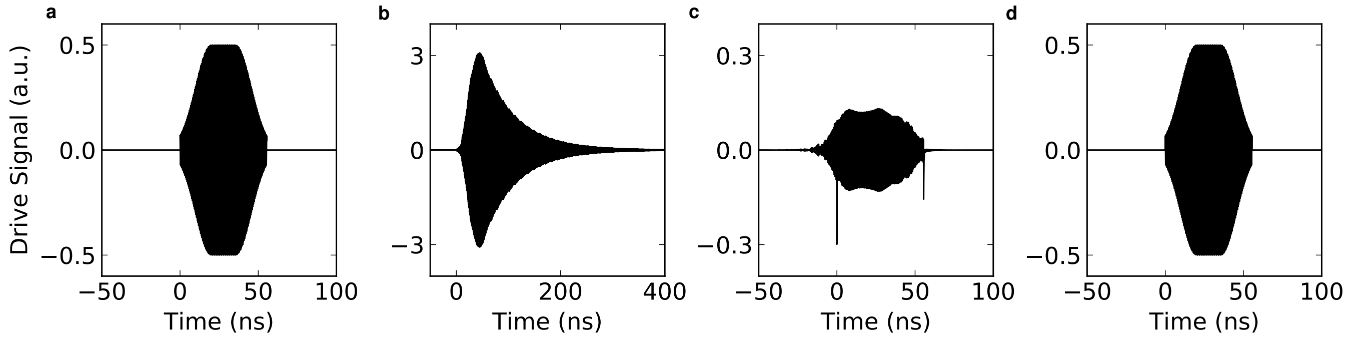

Supplementary Figure 6 | a, Ideal sideband pulse used perform a iSWAP between the transmon and mode 6. **b,** Expected distorted pulse at the location of the sample based on the measured transfer function of the flux line. **c,** Corrected AWG waveform calculated using Supplementary Equation (20). **d,** Expected pulse at the location of the sample due to distortion of the pulse in **c** through the flux line.

Supplementary Note 8 – Coupling between the transmon and the multimode memory

The rate of stimulated vacuum Rabi oscillations are related to the modulation strength and the bare coupling according to Supplementary Equation (10) of the main text. We can therefore extract the bare couplings from the measured sideband Rabi oscillation rates, particularly since the strength of the modulation can be independently calibrated from spectroscopy of the DC-offset of the transmon frequency (see Supplementary Note 7).

Instead, we measure the eigenmode-state dependent dispersive shift of the transmon frequency. The shift for each mode k is measured with a transmon Ramsey interference experiment conducted after loading a photon into mode k, according to the protocol shown in Supplementary Figure 7(a). The dispersive shift is related to the measured

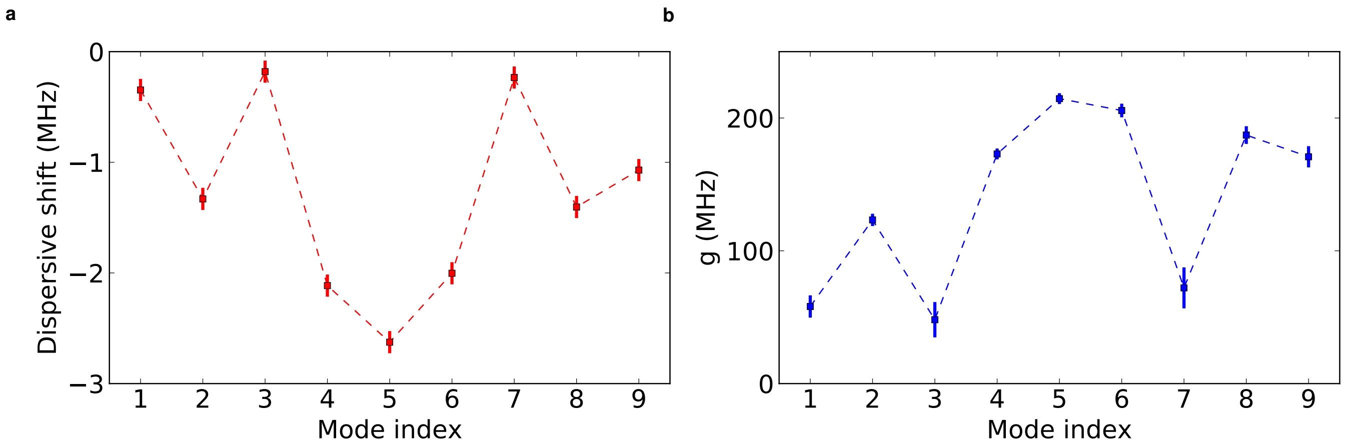

Supplementary Figure 7 | a, Dispersive shift χ_k as a function of mode index with the transmon biased at $\nu^{ge} = 4.3325$ GHz. The qubit frequency is stabilized to 50 kHz prior to each measurement and corresponds to the indicated error bar. **b,** The transmon-mode couplings g_k, extracted from the measured dispersive shift using equation (21), with the errors inferred from those in χ.

oscillation frequency $\nu_{\rm osc}$ and the Ramsey frequency ν_{Ram} according to $\delta_i = \nu_{\rm osc} - \nu_{\rm Ram}$, and is plotted as a function of mode number in Supplementary Figure 7(b). We extract the coupling rate g_k from the measured dispersive shift χ_k, which are related by [10]:

$$\chi_k = \frac{g_k^2 \alpha}{\Delta_k(\Delta_k + \alpha)}, \tag{21}$$

where α is the transmon anharmonicity and $\Delta_k = \nu_q - \nu_k$ is the detuning between the transmon and mode k. The g_k's extracted from this expression are shown in Supplementary Figure 7(c). The bare coupling rates extracted from the stimulated vacuum Rabi rate and from the dispersive shift are found to be consistent.

Supplementary Note 9 – Hamiltonian tomography

We use Hamiltonian tomography [11] to extract the $2N - 1$ parameters of a chain of N nearest-neighbour coupled resonators. We assume Hamiltonian for this chain is given by equation (1) of the main text, but allowing disorder of

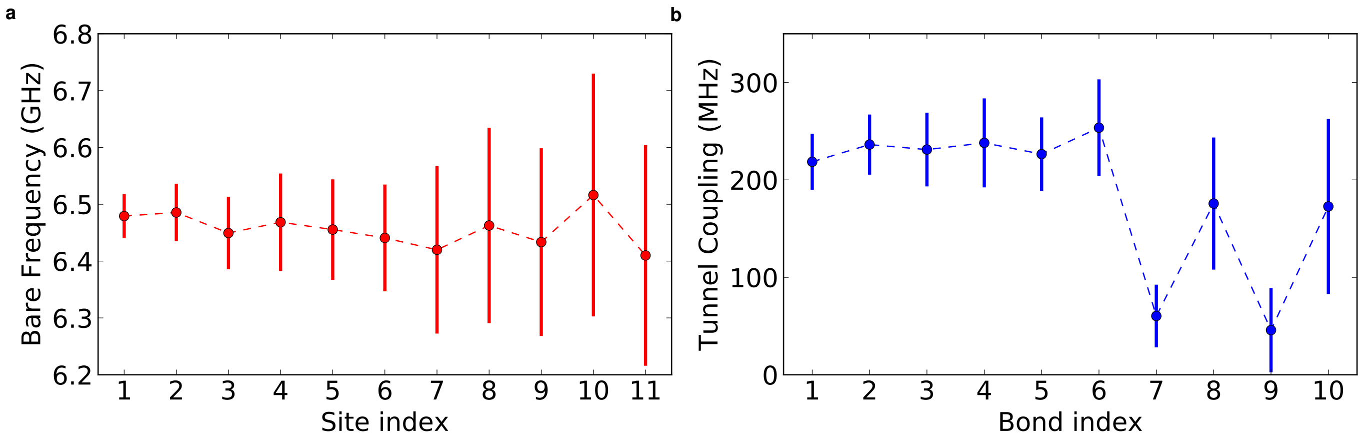

Supplementary Figure 8 | a, Bare resonator frequencies $\nu_{\mathrm{r},i}$ and **b,** nearest-neighbor tunnel couplings $g_{\mathrm{r},i}$ obtained from Hamiltonian tomography. The error bars in the bare frequencies and the couplings are obtained assuming a 25% uncertainty in the measured g_k's and a 0.5% uncertainty in the measured eigenfrequencies.

the individual resonator frequencies ($\nu_{\mathrm{r},i}$) and tunnel couplings ($g_{\mathrm{r},i}$):

$$\hat{H} = \sum_{i=1}^{N} \nu_{\mathrm{r},i} \hat{a}_i^\dagger \hat{a}_i + \sum_{i=1}^{N-1} g_{\mathrm{r},i} \left(\hat{a}_{i+1}^\dagger \hat{a}_i + \mathrm{c.c.} \right) \tag{22}$$

We extract these parameters using the frequencies (ν_k) and couplings to the transmon (g_k) of the eigenmodes of the array ($2N$ numbers). The coupling to the transmon is proportional to the amplitude of the memory-mode wavefunction at the edge resonator ($g_{\mathrm{eff},k} \propto |\phi_1^k|$), where the creation operator for eigenmode k is $\hat{b}_k^\dagger = \sum_i \phi_i^k \hat{a}_i^\dagger$. The bare frequencies and tunnel-couplings of the resonator are then extracted by iteratively solving the Schrödinger equation starting from the transmon end of the chain, while imposing the constraints from wavefunction normalization ($\sum_k \left(\phi_i^k\right) \left(\phi_j^k\right)^* = \delta_{ij}, \sum_i \left(\phi_i^k\right) \left(\phi_i^q\right)^* = \delta_{kq}$), as shown below:

$$\nu_{r,i} = \sum_k \nu_k \left|\phi_i^k\right|^2 \quad , \quad \phi_i^k = \frac{\left(\nu_k - \nu_{\mathrm{r},i-1}\right) \phi_{i-1}^k - g_{\mathrm{r},i-2} \phi_{i-2}^k}{g_{\mathrm{r},i-1}} \quad , \quad g_{r,i}^2 = \sum_k \left(\nu_k - \nu_{\mathrm{r},i}\right)^2 \left|\phi_i^k\right|^2 - g_{\mathrm{r},i-1}^2. \tag{23}$$

The bare frequencies and tunnel couplings thus extracted are shown in Supplementary Figure 8a and 8b. We infer that two of the normal modes (see Supplementary Figure 3) being extremely weakly coupled is likely a result of coupler 7 and 9 being defective.

Supplementary Note 10 – Coherence of the multimode memory

The coherence times of the memory modes are characterized through protocols analogous to those for the transmon, with the qubit pulses sandwiched with a pair of transmon-mode iSWAP pulses to transfer the quantum state between the transmon and the mode. The results of T_1 and T_2^* measurements for mode 1 of the memory are shown in Supplementary Figure 9a and b, with the coherence times for all the modes, summarized in Supplementary Figure 9c. The T_2^* is not found to be T_1 limited for many of the modes.

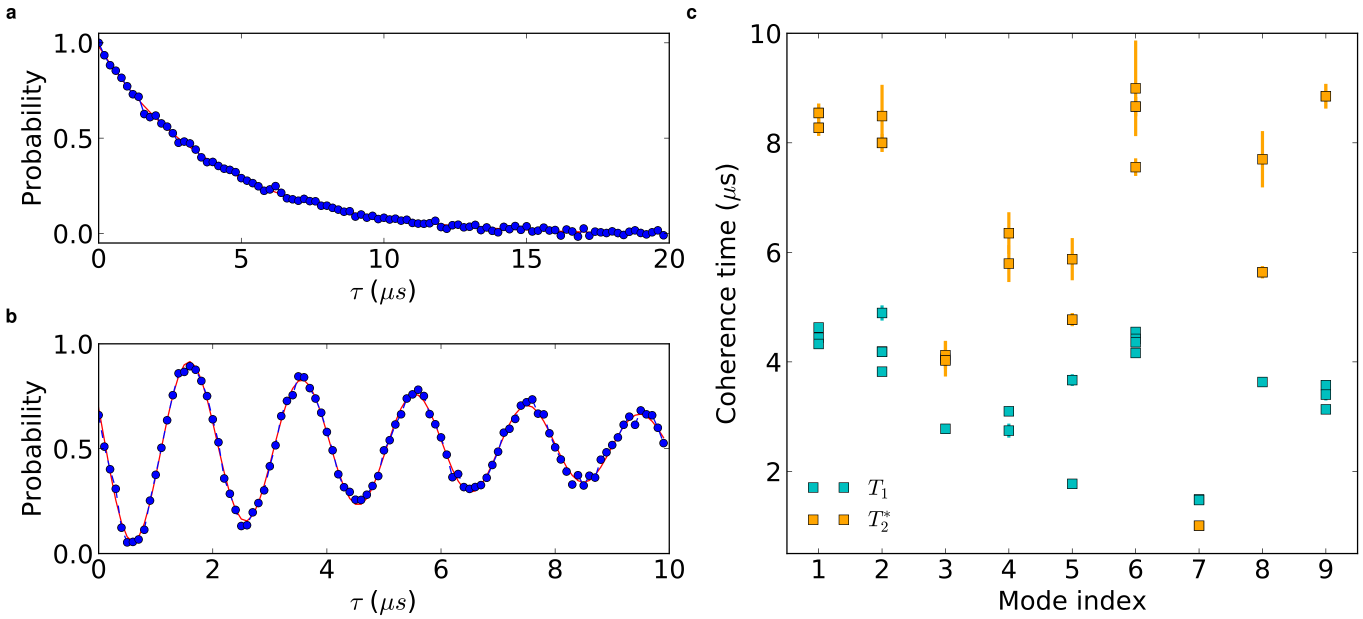

Supplementary Figure 9 | a, Single-photon lifetime measurement on mode 1. **b,** Ramsey (T_2^*) experiment on mode 1. The oscillation frequency of the Ramsey fringe can be used to infer the DC-shift of the transmon frequency during the iSWAP pulse. **c,** T_1 and T_2^* for the modes in the multimode memory. The error bars for individual points are as extracted from the fits. Because of insufficient statistics, the figure includes all the coherence data obtained with the transmon biased at $\nu_{\mathrm{q}} \sim 4.59$ GHz.

Supplementary Note 11 – Single-mode gate calibration

The frequency of the iSWAP pulse acting on a particular mode is obtained by choosing the frequency corresponding to maximum contrast of the stimulated vacuum Rabi chevrons, such as those in Supplementary Figure 3. The amplitude and pulse bandwidth of the flux-modulation pulses are optimized to maximize the oscillation rate, while minimizing cross talk with neighboring modes and sideband transitions across other transmon levels. The length of an iSWAP pulse is obtained using fits of stimulated vacuum Rabi oscillations such as in Figure 2c of the main text. To achieve high fidelity gate operations, we also calibrate and correct phase errors arising during the sideband pulses.

The main phase error in the flux-modulation pulse is due to the DC-shift of the transmon frequency during flux-modulation ($\delta\nu_{\mathrm{DC}}$ in equation (6)). The frequency of the center of the stimulated vacuum Rabi chevron is detuned from the difference frequency between the mode and the relevant transmon transition by $-\delta\nu_{\mathrm{DC}}$. Since the flux-pulse frequency is set to the center of the chevron, the clock (rotating frame) of the drive generator is shifted from the the frame of the Hamiltonian of equation 7. If the drive generator idles on resonance, there is an additional phase that accrues during that time. In the Ramsey experiment measuring the coherence time (T_2^*) of the modes (see Supplementary Figure 9a (top)), the accrued phase shifts the frequency of the Ramsey fringes by $\delta\nu_{\mathrm{DC}}$. We can then account for the misalignment of clocks by advancing the phase of the subsequent pulse by $2\pi\delta\nu_{\mathrm{DC}}\tau$. This correction can be easily implemented by keeping the drive clock aligned with the bare qubit-resonator system when the flux pulse is off, and incrementing the drive frequency by $\delta\nu_{\mathrm{DC}}$ during the iSWAP pulse to bring it into resonance with the DC-shifted frame.

Fixing the drive clock to be in sync with the Hamiltonian of Supplementary Equation (7) results in the absence of idle-time dependent phase errors. We additionally need to calibrate an additional dynamical phase (σ_z error) that occurs due to the change in the qubit frequency during the ramp up of the flux pulse. This phase is manifest in a rotating frame corresponding to the instantaneous qubit frequency $\bar{\nu}_{ge}(t)$ in equation (8). Repeating the transformation of equation (9) with a time-dependent qubit frequency results in an additional term:

$$\delta\hat{H} = -\frac{1}{2}h\frac{\partial\bar{\nu}_{ge}\left(t\right)}{\partial t}t\hat{\sigma}_z. \tag{24}$$

If we consider a square flux pulse with modulation amplitude corresponding to a DC-offset of ν_{DC} and pulse duration of t_π, the additional term in the Hamiltonian results in a dynamical phase of $\pi\nu_{\mathrm{DC}}t_\pi$. This error is calibrated using the sequence shown in Supplementary Figure 10a and corrected by adjusting the relative iSWAP pulse phases. The result of this calibration for one of the memory modes is shown in Supplementary Figure 10b. After calibrating the

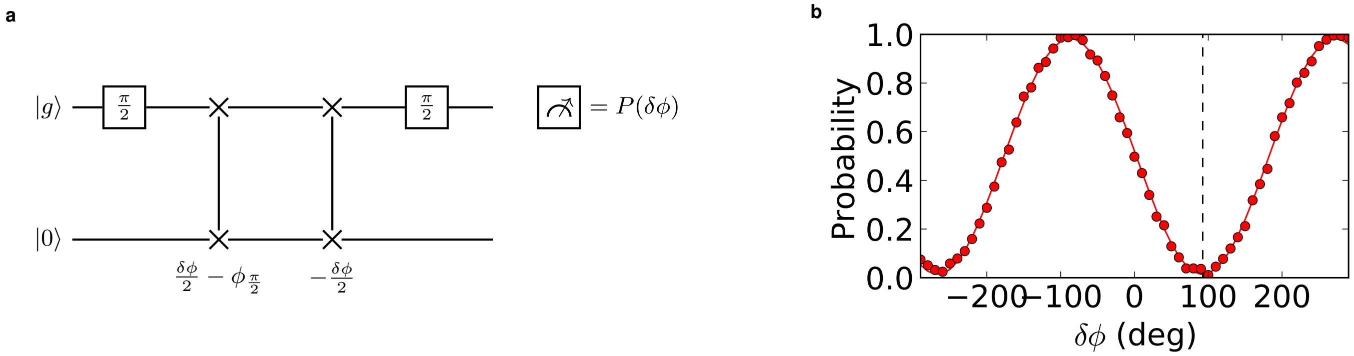

Supplementary Figure 10 | a, Circuit for calibrating phase of the iSWAP gate, where we sweep the phase $\frac{\delta\phi}{2}$ added and subtracted from the iSWAP pulses used to load and unload the state to the memory mode. We introduce an additional offset phase ($\phi_{\frac{\pi}{2}}$) which corrects σ_z errors occurring in the qubit pulse, ensuring that the mode state at the end of the first sideband pulse is free from all σ_z errors. The optimal phase for the iSWAP pulses is obtained by minimizing $P(\delta\phi)$. **b,** iSWAP phase calibration for mode 9 of the multimode memory, optimal phase ($92.5 \pm 0.1°$) indicated by the black dashed line.

iSWAP phase ($\phi^{\pi-\pi}$), we add (subtract) $\frac{\phi^{\pi-\pi}}{2}$ to every iSWAP pulse for loading (unloading) an excitation into each of the memory modes. Subsequent $|g\rangle - |e\rangle$ iSWAP pulses in all circuit diagrams include this phase correction, and are represented by $\tilde{\pi}$ when represented in an equation.

Supplementary Note 12 – Randomized benchmarking

We characterize the fidelity of single-mode operations using randomized benchmarking (RB) [12]. As described in the main text (see Figure 3), single-mode Clifford gates are realized by sandwiching single-qubit Clifford gates (C_i) with transmon-mode iSWAP pulses.

$$\tilde{C}_i = U_{\tilde{\pi}_{\rm sb}} C_i U_{\tilde{\pi}_{\rm sb}(\phi=\pi)} = C_i$$

To load the excitation to and from the transmon, we use σ_z error corrected sideband iSWAP pulses that are 180° out of phase with each other, so that the mode Cliffords are mapped directly from their transmon qubit counterparts. The Cliffords are generated by concatenating an operator each from $\{0, \frac{\pi}{2}_y, \pi_y, -\frac{\pi}{2}_y\}$ and $\{0, \frac{\pi}{2}_x, \pi_x, -\frac{\pi}{2}_x, \frac{\pi}{2}_z, -\frac{\pi}{2}_z\}$, to generate all 24 elements of the single qubit Clifford group. The circuit showing the sequence used for RB of the modes is shown in Supplementary Figure 11.

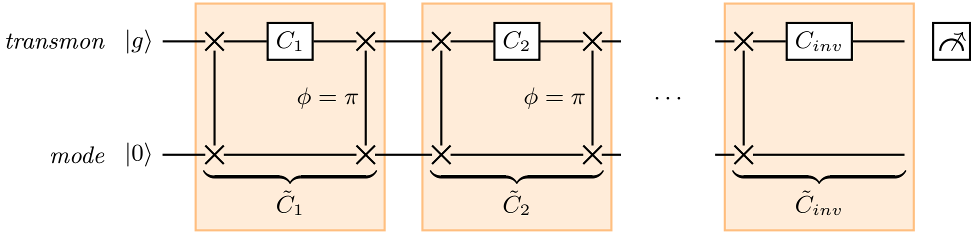

Supplementary Figure 11 | Randomized benchmarking (RB) characterizes the average random gate fidelity by acting random sequences of single-mode Clifford gates of increasing length, inverting the sequence, and then measuring the qubit state as a function of length. The sequence is acted on the multimode system initialized in the ground state, with the mode occupation error (ϵ) measured at the end of each sequence. The RB fidelity is extracted from the decay of the occupation fidelity ($1-\epsilon$) as a function of the length of the benchmarking sequence. For the data shown in Figure 3 of the main text, we use sequence lengths corresponding to those in [12, 13] and average over 32 random sequences.

The RB fidelity (p) is extracted by fitting the decay curves to the form $Ap^m + B$, where m is the sequence length. We estimate the coherence limit to the RB fidelity to be:

$$p_i = p_q - 2\left(1 - \exp\left[-\frac{t^{\pi}_{{\rm sb},k}}{T_{1,k}}\right]\right), \tag{25}$$

where $t^{\pi}_{sb,k}$ and $T_{1,k}$ are the iSWAP durations and lifetimes, respectively, of mode k, and p_q is the RB fidelity of the transmon. The experimentally measured fidelities approach these coherence limits (Figure 3 of the main text).

Supplementary Note 13 – Two mode gates using sideband transitions

The level diagram describing the relevant multimode states and transitions involved in the CZ gate are shown in Supplementary Figure 12a. We break the 2π $|e\rangle - |f\rangle$ sideband pulse used to provide a conditional phase between the transmon and the second mode ($|e1\rangle \to -|e1\rangle$, see Figure 4a of main text) into two π pulses. Control of the relative phases between these pulses allows for the correction of additional phase errors arising from the dispersive shift and the realization of an arbitrary controlled phase gate.

We obtain a CNOT gate by inserting an $|e\rangle - |f\rangle$ transmon charge π pulse ($\pi^{ef}_{\rm q}$) between the two $|e\rangle - |f\rangle$ sideband iSWAP pulses. This allows for mapping $|e0\rangle$ to $|e1\rangle$ (and vice versa) via the $|f0\rangle$ (qubit-mode CNOT), which again becomes a mode-mode CNOT gate when sandwiched with two $|g\rangle - |e\rangle$ sideband iSWAP pulses. The pulse sequence, energy level diagram, and relevant transitions for the CNOT gate are shown in Supplementary Figure 12b.

Slight modifications of these pulse sequences allow the realization of other two-mode gates such as the mode-mode CY and SWAP gates. The pulse sequences (without corrections from the dispersive shift) for realizing these two-mode gates are summarized in Supplementary Table 1.

Supplementary Note 14 – Phase error corrections to two-mode gates

The previous discussion of two-mode gates only involved resonant first-order sideband transitions and ideal transmon charge drive pulses. This idealized description is corrected by additional terms in the Hamiltonian of equation

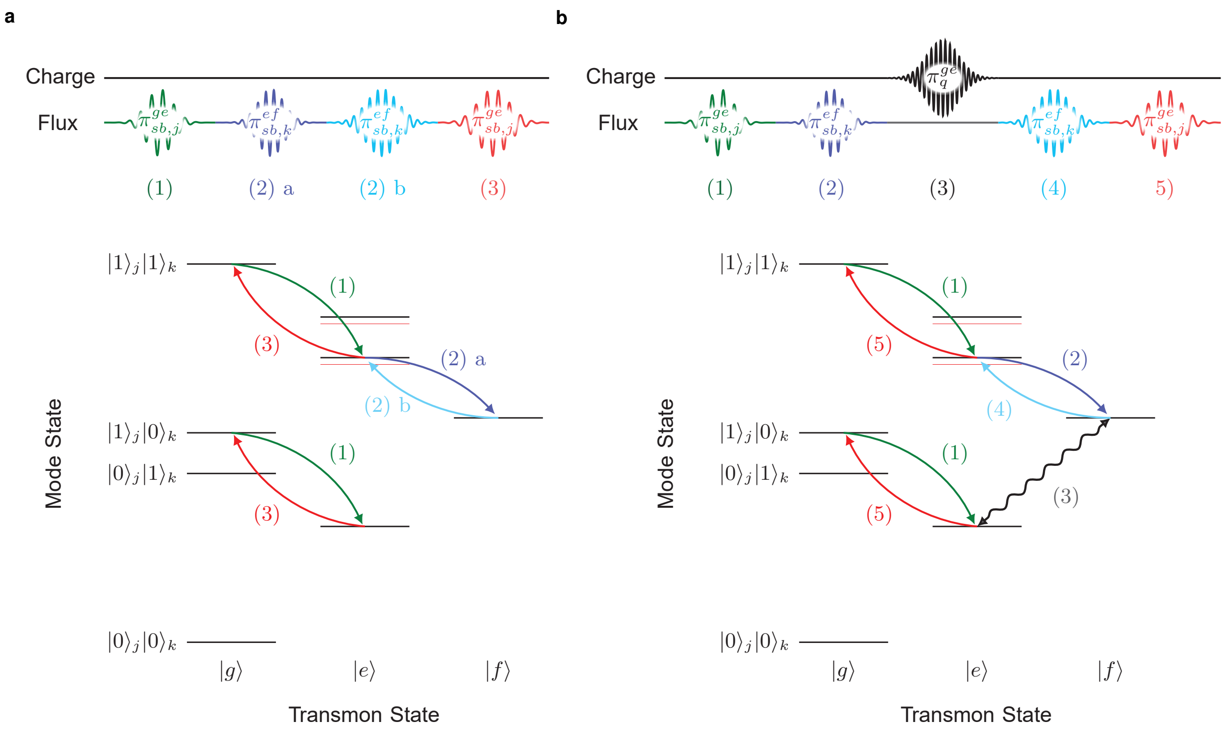

Supplementary Figure 12 | a and b, Energy level diagrams showing the multimode states and pulses involved in CZ and CNOT gates, respectively. The $e^{i\pi}$ phase factor arising from sideband SWAP operations between $|e01\rangle \longleftrightarrow |f00\rangle$ is modified by the dispersive shift (χ) of $|e01\rangle$ level (red). The additional phase arising from the dispersive shift is corrected by adjusting the phase of an ef sideband pulse on mode 1. CY gates are realized by adjusting the phase of the π_{q}^{ef} pulse.

Two-mode gate	Pulse Sequence
$\mathrm{CZ}_{\mathrm{j,k}}$	$\pi^{ge}_{\mathrm{sb},j} + \pi^{ef}_{\mathrm{sb},k} + \pi^{ef}_{\mathrm{sb},k} + \pi^{ge}_{\mathrm{sb},j}(\phi = \pi)$
$\mathrm{CX}_{\mathrm{j,k}}$	$\pi^{ge}_{\mathrm{sb},j} + \pi^{ef}_{\mathrm{sb},k} + \pi^{ef}_{\mathrm{q},y} + \pi^{ef}_{\mathrm{sb},k} + \pi^{ge}_{\mathrm{sb},j}(\phi = \pi)$
$\mathrm{CY}_{\mathrm{j,k}}$	$\pi^{ge}_{\mathrm{sb},j} + \pi^{ef}_{\mathrm{sb},k} + \pi^{ef}_{\mathrm{q},x} + \pi^{ef}_{\mathrm{sb},k} + \pi^{ge}_{\mathrm{sb},j}(\phi = \pi)$
$\mathrm{SWAP}_{\mathrm{j,k}}$	$\pi^{ge}_{\mathrm{sb},j} + \pi^{ef}_{\mathrm{sb},k} + \pi^{ge}_{\mathrm{sb},k} + \pi^{ef}_{\mathrm{sb},k} + \pi^{ge}_{\mathrm{sb},j}$

Supplementary Table 1 | Pulse sequences used for realizing various two-mode gates. j and k are indices corresponding to the control and target mode, respectively.

(11). The dominant additional effects are from: (1) dispersive shifts arising from photons in the multimode memory, (2) the qubit DC-offset due to flux modulation, and (3) phases from AC Stark shifts due to off-resonant first-order sidebands. These shifts result in corrections to the transmon rotation and transmon-mode iSWAP unitaries.

For the case of the dispersive shift, the corrections to the target unitaries depend on the quantum state of the multimode memory and result in a transmon-mode ZZ error. If we ignore photons in the rest of the memory, the effect of the dispersive shift on ther modes involved in a two-mode entangling gate can be inferred from the energy level diagram of Supplementary Figure 12. The dispersive shift causes the $|e10\rangle$ and $|e01\rangle$ levels to be shifted (red) from their bare values (black). As a result, sidebands resonant with $|e00\rangle \longleftrightarrow |g10\rangle$ are off-resonant from $|e01\rangle \longleftrightarrow |g11\rangle$.

The dispersive shift results in a phase and population error in the $|e01\rangle$ state. For the dispersive shifts χ and iSWAP times t_π used in this work, this population error is $\propto (\chi t_\pi)^2$ and, at worst, $\sim 5\%$ over the course of a CZ gate. This error is uncorrected and factors into the total gate error. The phase error on other hand is $\propto (\chi t_\pi)$ and results in a more significant effect (see Supplementary Figure 13a). Given that the $|e01\rangle$ state affected by the dispersive shift is selectively addressed by the $|e\rangle - |f\rangle$ sideband pulses used in the gate (see Supplementary Figure 12), this phase error

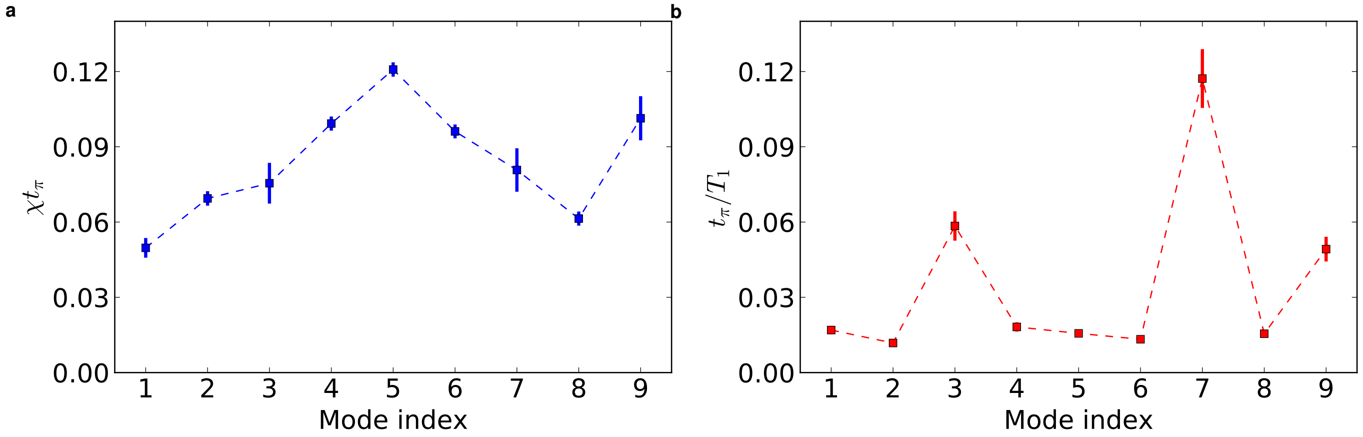

Supplementary Figure 13 | a, Error arising from the dispersive shift χ during an iSWAP pulse of duration t_π. The linear order phase errors ($\sim 25-60^\circ$ over the course of a CZ gate) are corrected during the gate, leaving residual amplitude error ($\propto (\chi t_\pi)^2$). The error bar in χt_π is dominated by the error in the measurement of χ. **b,** Error arising from T_1 loss during a sideband, plotted for comparison to the dispersive shift error. The $T_1's$ are the mean of the data in Supplemetary Figure 9, with the error in $\frac{t_\pi}{T_1}$ extracted from a 10% uncertainty in the T_1 of each mode.

is calibrated and corrected by adjusting the relative phase between these pulses. The experimental protocols used for the gate calibration and phase error correction for the CZ gate are described in Supplementary Note 15. The state dependent phases arising in the gate can be calculated by considering the effective Hamiltonian of equation (14) in the 8×8 subspace of levels relevant for the gates and shown in Supplementary Figure 12:

$$\tilde{H}(t) = \begin{pmatrix} 0 & 0 & 0 & 0 & \Omega_{ge}^* & 0 & 0 & 0 \\ 0 & 0 & 0 & 0 & g_{k,ge}^* & \Omega_{ge}^* & 0 & 0 \\ 0 & 0 & 0 & 0 & g_{j,ge}^* & 0 & \Omega_{ge}^* & 0 \\ 0 & 0 & 0 & 0 & 0 & g_{j,ge}^* & g_{k,ge}^* & 0 \\ \Omega_{ge} & g_{k,ge} & g_{j,ge} & 0 & 0 & 0 & 0 & \Omega_{ef}^* \\ 0 & \Omega_{ge} & 0 & g_{j,ge} & 0 & \delta_k & 0 & g_{k,ef}^* \\ 0 & 0 & \Omega_{ge} & g_{k,ge} & 0 & 0 & \delta_j & g_{j,ef}^* \\ 0 & 0 & 0 & 0 & \Omega_{ef} & g_{k,ef} & g_{j,ef} & 0 \end{pmatrix} \begin{matrix} |g\rangle\otimes|0_j0_k\rangle \\ |g\rangle\otimes|0_j1_k\rangle \\ |g\rangle\otimes|1_j0_k\rangle \\ |g\rangle\otimes|1_j1_k\rangle \\ |e\rangle\otimes|0_j0_k\rangle \\ |e\rangle\otimes|0_j1_k\rangle \\ |e\rangle\otimes|1_j0_k\rangle \\ |f\rangle\otimes|0_j0_k\rangle \end{matrix} \tag{26}$$

Here, the multimode state is labeled $|n_j, n_k\rangle$ and the phases of equation (16) have been absorbed into the g's and Ω's (which are time dependent), i.e.,

$$g_{i,\alpha} \to g_{i,\alpha}e^{i\phi_{\mathrm{sb},\alpha}} \quad , \quad \Omega_\alpha \to \Omega_\alpha e^{-i\phi_{\mathrm{q},\alpha}} \quad , \quad \alpha \in \{ge, ef\} \quad , \quad i \in \{j,k\}\,. \tag{27}$$

The $|e\rangle$–$|f\rangle$ sideband pulses act only on one transition and are unaffected by the state dependent shift when considering only two modes. We chose the $|e\rangle-|f\rangle$ sideband freqeuncy to be resonant with the $|f00\rangle$ and the dispersively shifted $|e01\rangle$ level. In the rotating frame of equation (26), this corresponds to $|e\rangle-|f\rangle$ first-order sidebands acquiring the following time-dependence:

$$g_{j,ef}(t) = \tilde{g}_{j,ef}e^{-2\pi i\delta_k t} \quad , \quad g_{k,ef}(t) = \tilde{g}_{k,ef}e^{-2\pi i\delta_j t}. \tag{28}$$

$\tilde{g}_{i,ef}$ is proportional to the envelope of the $|e\rangle-|f\rangle$ sideband pulse, and δ_k and δ_j are the dispersive shifts of $|e0_j1_k\rangle$ and $|e1_j0_k\rangle$ respectively.

We compute the action of the CZ and CNOT gate sequences by evolving the Hamiltonian above, with time dependent coefficients and phases as per Supplementary Table 2. In these pulse sequences, only one of the drive terms is on at any given time and the corresponding unitaries obtained upon integration of the Schrödinger equation are generalizations of those in Supplementary Equation (18), with corrections arising from the dispersive shift. The effective unitary thus

Gate	Pulse Sequence
CZ	$\tilde{\pi}^{ge}_{\mathrm{sb},j}(\phi_\mathrm{a}) + \tilde{\pi}^{ef}_{\mathrm{sb},k}(\phi_\mathrm{b}) + \tilde{\pi}^{ef}_{sb,k}(\phi_\mathrm{c}) + \tilde{\pi}^{ge}_{\mathrm{sb},j}(\phi_\mathrm{d})$
CNOT	$\tilde{\pi}^{ge}_{\mathrm{sb},j}(\phi_\mathrm{a}) + \tilde{\pi}^{ef}_{\mathrm{sb},k}(\phi_\mathrm{b}) + \pi^{ef}_{q}(\phi_\mathrm{e}) + \tilde{\pi}^{ef}_{\mathrm{sb},k}(\phi_\mathrm{c}) + \tilde{\pi}^{ge}_{\mathrm{sb},j}(\phi_\mathrm{d})$

Supplementary Table 2 | Nomenclature for the pulse phases used in the CZ and CNOT gates resulting in the unitary operators in equation (30).

realized for the CZ and CNOT gates, to lowest order in χ/g_sb and χ/Ω are;

$$\mathrm{U_{CZ}} = \begin{pmatrix} 1 & 0 & 0 & 0 \\ 0 & 1 & 0 & 0 \\ 0 & 0 & -e^{i(\phi_\mathrm{a}-\phi_\mathrm{d})} & 0 \\ 0 & 0 & 0 & e^{i\left(\phi_\mathrm{a}-\phi_\mathrm{d}+\phi_\mathrm{b}-\phi_\mathrm{c}-2\pi t^{\pi}_{\mathrm{sb},j,ge}\delta_k\right)} \end{pmatrix} \tag{29}$$

$$\mathrm{U_{CNOT}} = \begin{pmatrix} 1 & 0 & 0 & 0 \\ 0 & 1 & 0 & 0 \\ 0 & 0 & 0 & e^{i\left(\phi_\mathrm{a}-\phi_\mathrm{d}+\phi_\mathrm{b}+\phi_\mathrm{e}-\pi t^{\pi}_{\mathrm{sb},j,ge}\delta_k\right)} \\ 0 & 0 & e^{i\left(\phi_\mathrm{a}-\phi_\mathrm{d}-\phi_\mathrm{c}-\phi_\mathrm{e}-\pi t^{\pi}_{\mathrm{sb},j,ge}\delta_k\right)} & 0 \end{pmatrix} \tag{30}$$

We see that one can choose sideband pulse phases that cancel phases arising from the dispersive shift and thereby realize the target unitaries for the CZ and CNOT gates.

Supplementary Note 15 – CZ gate phase calibration sequences

Here, we describe protocols used to calibrate and correct each of the additional phases arising in the CZ gate. The phases of the iSWAP pulses used in the CZ gate are defined below, where $\phi_{1,2}$ are the controlled phases:

$$CZ_{j,k}(\phi_1, \phi_2) = \tilde{\pi}^{ge}_{\mathrm{sb},j} + \pi^{ef}_{\mathrm{sb},k}(\phi_1) + \pi^{ef}_{\mathrm{sb},k}(0) + \tilde{\pi}^{ge}_{\mathrm{sb},j}(\phi_2). \tag{31}$$

j is the control mode and k is the target mode of the CZ gate, with the states labeled $|n_j, n_k\rangle$, and $\tilde{\pi}$ indicating iSWAP pulses for which the DC-offset σ_z error is corrected within the pulse. From equation (29), we see that only two relative phase adjustments ($\phi_\mathrm{a} - \phi_\mathrm{d}$ and $\phi_\mathrm{b} - \phi_\mathrm{c}$) are required to correct the dispersive shift error. Here, we adjust these relative phases by controlling $\phi_1 = \phi_\mathrm{b}$ and $\phi_1 = \phi_\mathrm{d}$, while leaving ϕ_a and ϕ_c fixed. We measure each phase error through Ramsey experiments with initial states that are appropriate superpositions of the basis states, as indicated in Supplementary Table 3.

Sequence	Phase error	Initial state	# CZ's	Measured mode
a	Dispersive shift (SPAM)	$\|10\rangle + \|11\rangle$	1	Target (k)
b	Dispersive shift (Gate)	$\|10\rangle + \|11\rangle$	2	Target (k)
c	DC-offset during gate	$\|01\rangle + \|11\rangle$	1	Control (j)
d	Off-resonant sidebands	$\|00\rangle + \|01\rangle$	1	Target (k)

Supplementary Table 3 | Summary of CZ gate phase calibration experiments that correct for each of the sources of the phase errors. The initial states used to calibrate each of the phase errors are indicated.

The phase error from the dispersive shift is obtained by preparing the system in the state $|\psi_p\rangle = |10\rangle + |11\rangle$. We measure the relative phase between the basis states after applying the CZ gate, using the sequence in Figure 14a. The dispersive shift results in the $|11\rangle$ acquiring an additional phase in the preparation (ϕ_p), gate (ϕ_g) and measurement segments (ϕ_m). Similar additional phases also accrue during the gate (ϕ_g) and the measurement (ϕ_m) segments. We sweep the phase ($\delta\phi$) of the first $|e\rangle - |f\rangle$ sideband pulse of the CZ gate (see Supplementary Equation (31)). The phase that *maximizes* the final measured transmon population provides the total added phase, $\phi^\mathrm{ds}_\mathrm{t} = \phi_\mathrm{p} + \phi_\mathrm{g} + \phi_\mathrm{m}$. $CZ_{j,k}(\phi^\mathrm{ds}_\mathrm{t}, 0)$ is a combination of an ideal CZ gate and the $C\phi$ gate that cancels additional phases arising from the dispersive shift over the entire sequence. We isolate the state-dependent phase error arising only during the CZ gate by adding a second Cϕ gate to the previous sequence, as shown in Supplementary Figure 14b. We sweep the phase

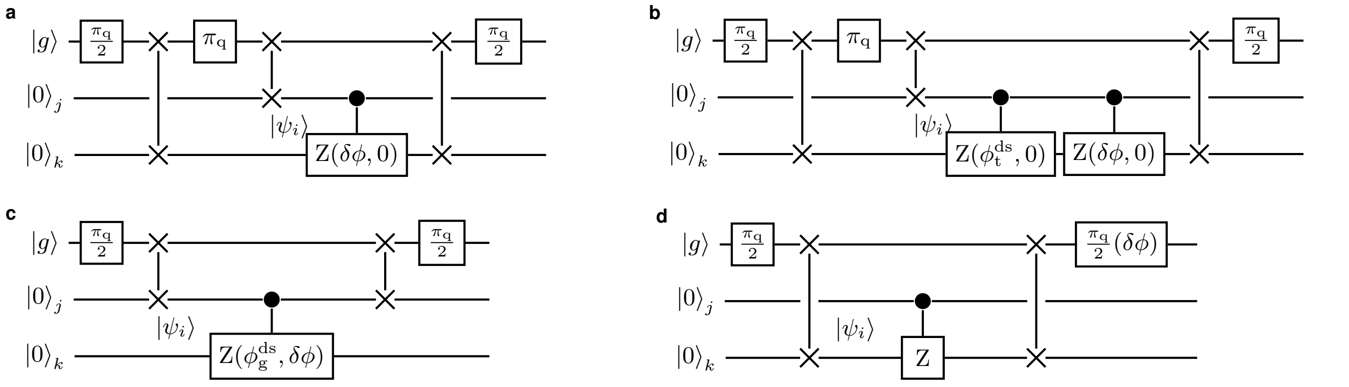

Supplementary Figure 14 | CZ gate calibration sequences. a, Measures phase error from the dispersive shift arising from the entire sequence. **b,** Isolates the error from the dispersive shift error occuring only during the gate. **c,** Measures the phase error arising from the qubit dc-offset occuring during the gate. **d,** Phase error arising from the AC Stark shift due to off-resonant first order sidebands.

$(\delta\phi)$ of the (first) $|e\rangle - |f\rangle$ sideband pulse of the second Cϕ gate, with $\phi_1 = \phi_{\rm ds}^{\rm t}$ for the first Cϕ gate. Given the same preparation and measurement sequences, the SPAM phases are corrected by construction by the first Cϕ gate. We find the phase $\delta\phi$ that *minimizes* the population of the transmon, thus realizing a CZ gate that flips the sign of the $|11\rangle$ state. The $CZ_{k,j}(\phi_{\rm g}, 0)$ gate therefore is corrected for phases from dispersive shifts occurring during the gate sequence.

We obtain a fully corrected CZ gate by correcting the relative phase between the $\{|00\rangle, |01\rangle\}$ and $\{|10\rangle, |11\rangle\}$ manifolds. These state manifolds have a relative phase resulting from the transmon frequency DC-offset occurring during the $|e\rangle - |f\rangle$ sidebands of the CZ gate. We correct this additional phase by adjusting the phase of the final $|g\rangle - |e\rangle$ sideband pulse of the CZ gate (ϕ_2 in equation (31)), using the experimental sequence of Supplementary Figure 14c. The resulting $\rm CZ_{j,k}(\phi_g^{ds}, \phi^{DC})$ gate is therefore corrected of errors from dispersive shifts and qubit flux-modulation DC-offsets.

The phase error resulting from dispersive shifts due to off-resonant first-order sidebands are measured by acting the CZ gate on the $|00\rangle + |01\rangle$ state. The CZ gate nominally does not change this state, and we correct this phase error using subsequent qubit pulses as shown in Supplementary Figure 14d. This phase is significant only for gates between modes with spectral spacing near the anharmonicity of the transmon.

Supplementary Note 16 – Two-mode quantum state tomography

Reconstructing the density matrix of an arbitrary two-qubit state requires the measurement of all possible two-qubit correlations $\{\langle \rm XI\rangle, \langle XX\rangle \ldots \langle ZZ\rangle\}$, i.e.;

$$C_{i,j} = \langle B_i \otimes B_j\rangle \;\;|\;\; B_i \in \{\rm I, X, Y, Z\} \tag{32}$$

These correlators can be measured through Ramsey interferometry, as described in the main text [14]. We equivalently measure all the necessary correlations with the aid of the single and two-mode gate operations prior to measuring the state of the transmon. A sideband iSWAP pulse ($\pi_{\rm sb}$) on the $|g\rangle - |e\rangle$ transition, along with single qubit rotations alone can be used to measure all single-mode correlators $C_{ij} \in \{\langle B_i \otimes I\rangle \text{ or } \langle I \otimes B_i\rangle\} \mid B_i \in \{\rm X, Y, Z\}$.

The entanglement information is present in two-mode correlators, $C_{i,j} = \langle \rm B_i \otimes B_j\rangle \mid B_i \in \{X, Y, Z\}$. We measure these correlators by acting two-mode gates before measuring a single-mode correlators. For instance, the $\langle XX\rangle$ correlator of a given state ($|\psi_i\rangle$) is measured by acting CX gate prior to the measurement of $\langle \rm XI\rangle$. In the Heisenberg picture [15], the transformation is shown below:

$$C = \langle\psi_f| X \otimes I |\psi_f\rangle = \langle\psi_i| U_{\rm CX}^{\dagger} (X \otimes I) U_{\rm CX} |\psi_i\rangle = \langle\psi_i| X \otimes X |\psi_i\rangle \tag{33}$$

Here $|\psi_i\rangle$ is the two-mode state to be measured and $|\psi_f\rangle = U_{\rm CX}|\psi_f\rangle$ is the state obtained following action of the CX gate. A summary of pulse sequences used for the measurement of each of the correlations required for two-mode tomography are shown in Table 4.

We extract the correlators and construct the density matrix of the two-mode state from the measured transmon

#	Measured Correlation	Pulse Sequence
0	$-\langle \mathrm{IX} \rangle$	$\pi_{\mathrm{sb},k} + \frac{\pi}{2}_y$
1	$\langle \mathrm{IY} \rangle$	$\pi_{\mathrm{sb},k} + \frac{\pi}{2}_x$
2	$\langle \mathrm{IZ} \rangle$	$\pi_{\mathrm{sb},k}$
3	$-\langle \mathrm{XI} \rangle$	$\pi_{\mathrm{sb},j} + \frac{\pi}{2}_y$
4	$-\langle \mathrm{XX} \rangle$	$\mathrm{CX} + \pi_{\mathrm{sb},j} + \frac{\pi}{2}_y$
5	$-\langle \mathrm{XY} \rangle$	$\mathrm{CY} + \pi_{\mathrm{sb},j} + \frac{\pi}{2}_y$
6	$\langle \mathrm{XZ} \rangle$	$\mathrm{CZ} + \pi_{\mathrm{sb},j} + \frac{\pi}{2}_y$
7	$\langle \mathrm{YI} \rangle$	$\pi_{\mathrm{sb},j} + \frac{\pi}{2}_y$
8	$\langle \mathrm{YX} \rangle$	$\mathrm{CX} + \pi_{\mathrm{sb},j} + \frac{\pi}{2}_x$
9	$\langle \mathrm{YY} \rangle$	$\mathrm{CY} + \pi_{\mathrm{sb},j} + \frac{\pi}{2}_x$
10	$-\langle \mathrm{YZ} \rangle$	$\mathrm{CZ} + \pi_{\mathrm{sb},j} + \frac{\pi}{2}_x$
11	$\langle \mathrm{ZI} \rangle$	$\pi_{\mathrm{sb},1}$
12	$-\langle \mathrm{ZX} \rangle$	$\mathrm{CZ} + \pi_{\mathrm{sb},k} + \frac{\pi}{2}_y$
13	$\langle \mathrm{ZY} \rangle$	$\mathrm{CZ} + \pi_{\mathrm{sb},k} + \frac{\pi}{2}_x$
14	$\langle \mathrm{ZZ} \rangle$	$\mathrm{CX} + \pi_{\mathrm{sb},k}$

Supplementary Table 4 | Pulse sequences used for the measurement of all two-qubit correlations between mode pairs. These correlations are used to reconstruct a two-qubit density matrix using equation 34.

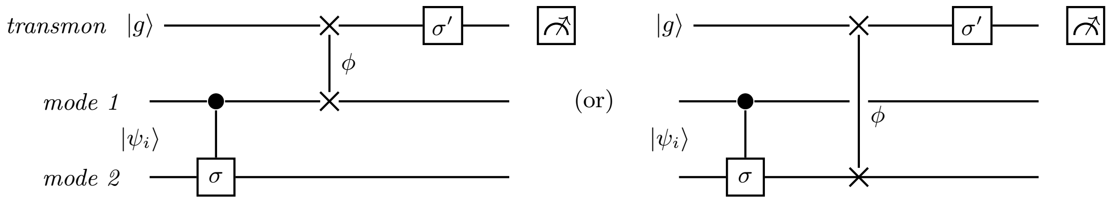

Supplementary Figure 15 | General two-mode correlator measurement sequence, where $\sigma, \sigma' \in \{\mathrm{I}, \mathrm{X}, \mathrm{Y}, \mathrm{Z}\}$. To measure and correct additional phase shifts (as described in Supplementary Note 15) arising in the tomography sequence, we sweep the phase, ϕ, of the final sideband pulse of the sequence used to measure each correlator.

population P_{ij} at the end of the sequence for each correlator C_{ij} using;

$$C_{i,j} = \langle B_i \otimes B_j \rangle = 2P_{ij} - 1,$$
$$\rho = \sum_{ij} \frac{C_{ij} B_i \otimes B_j}{4}. \tag{34}$$

In general, fast measurement and reset [16] of the transmon would allow us to perform sequential measurements of two-mode correlations using the transmon without requiring mode-entangling gate operations. For each mode, we would map the mode state to the transmon with an iSWAP, measure the transmon, and reset it to the ground state. The transmon state could be reset with an iSWAP back to the measured mode or to an auxiliary mode. The transmon can subsequently be used to measure the next mode. Additionally, we can perform Wigner tomography [17] of the multimode chain through direct measurements of the multimode fields and parametric amplification. These techniques pose more stringent conditions on the measurement fidelity and speed and are beyond the scope of this work.

We account for spurious phases arising in the Bell state tomography sequence by varying the phase of the final sideband pulse used in each correlator measurement. The results of such phase sweeps for the $|\Phi^+\rangle$ and $|\Psi^+\rangle$ Bell states are shown in Supplementary Figure 20a and b. We note that for these states, the only correlators that are functions of the final sideband phase are $\mathrm{XX}, \mathrm{XY}, \mathrm{YX}, \mathrm{YY}$ and the $|\Phi^+\rangle$ and $|\Psi^+\rangle$ Bell states give opposite answers for measurements of the two-mode parity ZZ. The optimal sideband phase that accounts for the additional phases shifts are indicated by the dashed lines. We extract the density matrices from the results of these measurements using

equation (34). The state fidelities for the two states are calculated from the overlap of the ideal ρ_{id} and measured ρ_{m} density matrices: $F_p = \text{Tr}(\rho_{\text{id}}\rho_{\text{m}})$.

Supplementary Note 17 – Process tomography of two-mode gates

Process tomography of a two-qubit gate consists of quantum state tomography after acting the gate on a set of 16 linearly independent input states that form a basis for representing an arbitrary two-qubit density matrix [18]. Process tomography of two-mode gates therefore consists of a set of 240 measurement sequences of the form shown in Supplementary Figure 16.

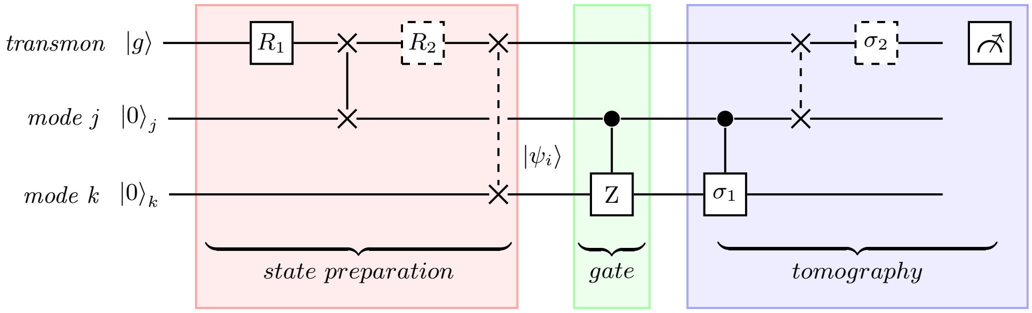

Supplementary Figure 16 | Process tomography sequence for two-mode gates, broken down into preparation (red), gate (green), and tomography (blue) segments. For the preparation sequence, we use qubit rotations $R_{1,2} = \left\{I, R_y\left(\frac{\pi}{2}\right), R_x\left(\frac{\pi}{2}\right), R_x(\pi)\right\}$ and DC-offset corrected sideband iSWAP pulses with an additional $-\frac{\pi}{2}$ phase, such that the target multimode state at the end of the preparation sequence is $|\psi_i\rangle = R_1 \otimes R_2 |0_j 0_k\rangle$. We measure the density matrix of the gate outputs for given input density matrices $\rho_i = |\psi_i\rangle\langle\psi_i|$ using the state tomography protocols of Supplementary Note 16 and the sequences in Supplementary Table 4, corresponding to $\sigma_{1,2} = \{I, X, Y, Z\}$ in the sequence shown above. We note that the iSWAP gate acts on mode k for some of the correlators, and that the tomography sequences that measure single-mode correlators have no additional two-mode gate, corresponding to $\sigma_1 = \mathbb{1}$. The qubit and iSWAP operations that are indicated by the dashed lines have errors arising from the dispersive shift.

The gate calibration protocols described in Supplementary Note 15 for the CZ gate, and analogous protocols for the CX gate, correct phase errors due to dispersive shifts and the transmon DC-offset from flux modulation during the gate. We additionally correct errors arising from the dispersive shift during the state preparation and tomography (SPAM) segments of the various process tomography sequences. These errors occur in the qubit and iSWAP operations indicated by dashed lines in Supplementary Figure 16. The dispersive shift causes amplitude and phase errors in the transmon and sideband pulses. We again correct only phase errors to first-order in χ/Ω_{sb}. These controlled-phase errors can be formally incorporated as $\text{C}\Phi$ gates at the end of the preparation sequence ($\text{C}\Phi_{\text{p}}$) and prior the to measurement sequence ($\text{C}\Phi_{\text{m}}$). These additional gates are concatenated into the gate and tomography sequences as shown in Supplementary Figure 17.

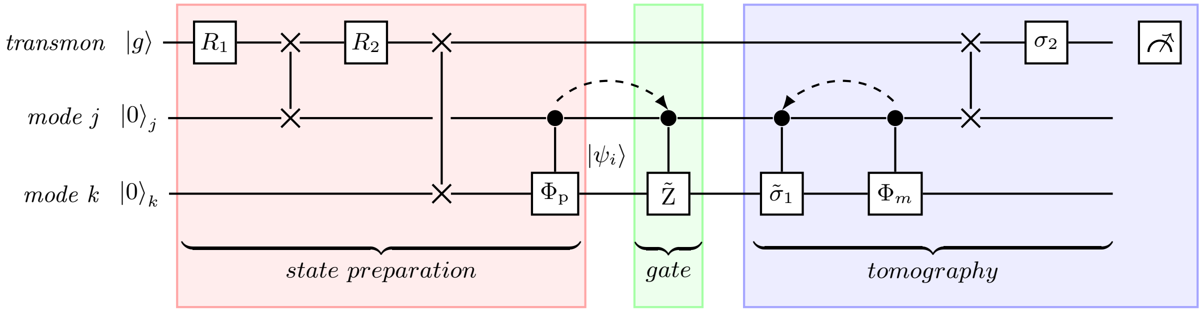

Supplementary Figure 17 | Protocol for correcting errors from the dispersive shift, in state preparation and measurement during process tomography of multimode gates.

$\text{C}\tilde{\text{Z}}$ and $\text{C}\tilde{\sigma}_1$ are chosen to give phase-corrected CZ and $\text{C}\sigma_1$ gates when concatenated with $\text{C}\Phi_{\text{p}}$ and $\text{C}\Phi_{\text{m}}$, respectively. The preparation error is corrected through an added phase (ϕ_p) in the first $|e\rangle - |f\rangle$ sideband of the first gate, while the tomography error is corrected through an added phase (ϕ_m) in the second $|e\rangle - |f\rangle$ sideband of the last gate

of the sequence. We thereby correct the sequence to first-order in the dispersive-shift error. The sideband phases are chosen in this manner in order to correct errors in both the $|10\rangle$ and $|11\rangle$ states (see equation (29), (30)). The phase errors depend on the duration of the qubit and sideband pulses used in the sequence. In the absence of loss, they can be calculated based on the dispersive shift and pulse shapes.

We calibrate the additional phase errors through process tomography of the Identity ($\mathbb{1}$) gate (idling for 10 ns). We find the optimal phases by sweeping the added controlled-phases of the Cϕ and Cσ gates, and comparing results for corresponding correlators with and without CX/CY gates (such as XX and XI, respectively) as shown in Supplementary Figure 18.

This scheme allows us to isolate state preparation and measurement errors (ϕ_p and ϕ_m). In the Heisenberg picture, working backward from the transmon measurement, we consider how correlators are modified by the dispersive shift and the correcting two-mode gates.

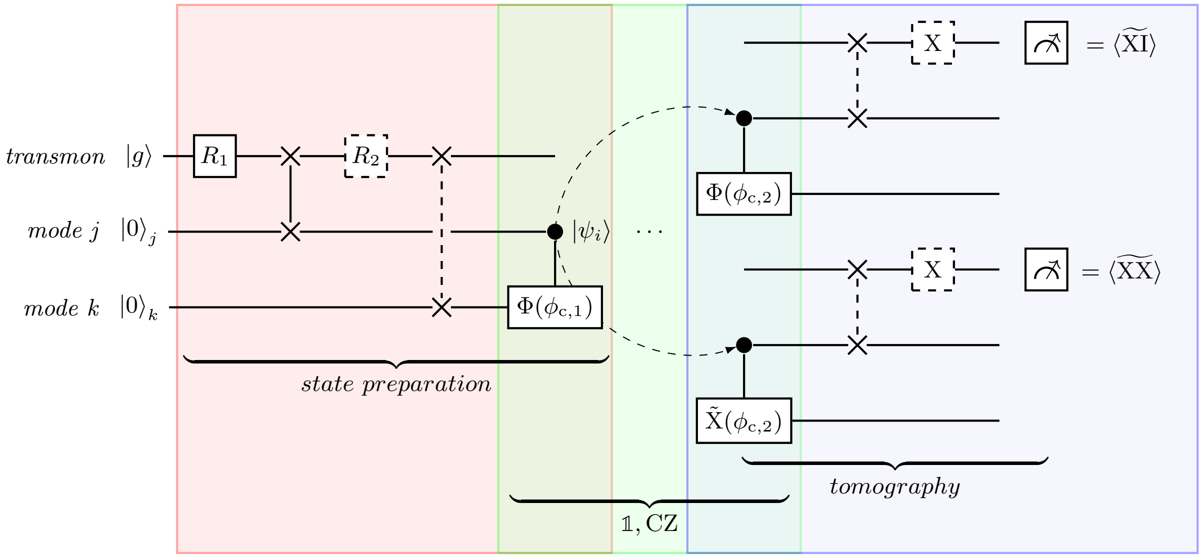

Supplementary Figure 18 | Process tomography of the $\mathbb{1}$ gate, used to calibrate the additional SPAM phase errors. We measuring the added phases to the $|10\rangle$ and $|11\rangle$ states by comparing the results of correlators with and without CNOT gates, thereby isolating state preparation and measurement errors.

As an example, for the prepared state $|\hat{x}\hat{x}\rangle$ ($R_{1,2} = Y_{\frac{\pi}{2}}$), the expected values of the correlators XI and XX are:

$$\langle\widetilde{\mathrm{XI}}\rangle = \cos^2\left(\frac{\phi_{\mathrm{c},1} + \phi_{\mathrm{c},2} - \phi_{\mathrm{p}} - \phi_{\mathrm{m}}}{2}\right), \tag{35}$$

$$\langle\widetilde{\mathrm{XX}}\rangle = \cos\left(\frac{\phi_{\mathrm{c},2} - \phi_{\mathrm{c},1} - \phi_{\mathrm{p}} - \phi_{\mathrm{m}}}{2}\right)\cos\left(\frac{\phi_{\mathrm{c},2} + \phi_{\mathrm{c},1} + \phi_{\mathrm{p}} - \phi_{\mathrm{m}}}{2}\right), \tag{36}$$

where ϕ_{p} and ϕ_{m} are the phase errors of the $|11\rangle$ state (relative to the other computational basis states) in state preparation and measurement, respectively. Finding and correcting ϕ_{p} and ϕ_{m} amounts to choosing $\phi_{\mathrm{c},1}$ and $\phi_{\mathrm{c},2}$ such that $\langle\widetilde{\mathrm{XI}}\rangle = \langle\widetilde{\mathrm{XX}}\rangle = 1$.

The additional phases only depend on the shape of the qubit and sideband pulse waveforms. As a result, we can calibrate ϕ_{p} and ϕ_{m} for all 240 sequences using a total of 13 unique experiments. We can then extract the full process matrix by measuring at the optimal angles obtained from the calibration experiments. We check that the validity of the calibrations by also additionally sweeping the phase of the final sideband pulse. In order to reduce SPAM error from decoherence, we combine the state preparation and measurement correction gates (as indicated by the arrows in Supplementary Figure 18) during process tomography of the $\mathbb{1}$ and CZ gates, noting that C$\Phi_{\phi_{c,1}}$ commutes with both of them.

We perform process tomography of the CZ gate by inserting it in place of the $\mathbb{1}$ in Supplementary Figure 18, after calibrating the tomography axes. A two-mode gate is fully characterized by the completely positive map $\mathcal{E}$;

$$\mathcal{E}(\rho) = \sum_{m,n=0}^{d^2-1} \chi_{mn}\hat{A}_m\rho\hat{A}_n^{\dagger}. \tag{37}$$

$\hat{A}_m = \hat{B}_i \otimes \hat{B}_j$, with $\hat{B}_i \in \{I, X, Y, Z\}$, forms a basis of operators acting on a two-mode state ρ. χ_{mn} is the process matrix characterizing the two-mode gate, and is extracted from the measured output density matrices ρ_j^{out} for 16 linearly independent input density matrices (ρ^j) as shown below:

$$\rho_j^{\text{out}} = \mathcal{E}(\rho_j) = \sum_k \lambda_{jk}\rho_k = \sum_{m,n} \chi_{mn}\hat{A}_m \rho \hat{A}_n^\dagger = \sum_{m,n,k} \chi_{mn}\beta_{jk}^{mn}\rho_k, \tag{38}$$

$$\Rightarrow \lambda_{jk} = \text{Tr}[\rho_k \rho_j^{\text{out}}] = \sum_{mn} \beta_{jk}^{mn}\alpha_{mn}. \tag{39}$$

Supplementary Equation (39) is directly inverted to obtain the process matrix α_{mn}. We do not impose the completeness condition, $\sum_{mn} \chi_{mn}\hat{A}_m\hat{A}_n^\dagger = \mathbb{1}$ as a constraint. This constraint arises from the probabilities of states in the relevant two-mode space summing to 1. This is not necessarily the case when there are several memory modes. The process fidelities are extracted from the measured (χ^{m}) and ideal process matrices (χ^{t}) using $F_p = \text{Tr}\left[\chi^{\text{m}}\chi^{\text{t}}\right]$.

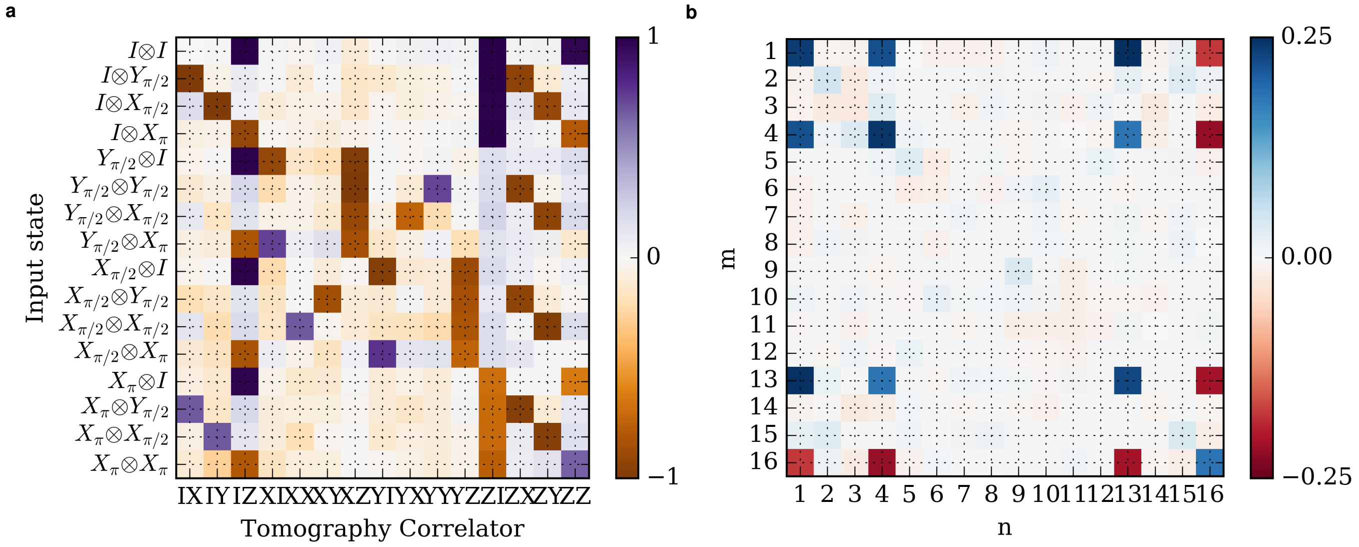

Supplementary Figure 19 | a, Experimentally measured correlators after correcting for phase errors arising during state preparation and measurement for process tomography of the CZ gate between mode $j = 2$ and $k = 6$. **b,** Process matrix extracted from the resulting measurements by inverting equation (39).

Supplementary Note 18 – Preparation of entangled states

We use a slight variant of the scheme described in the main text to prepare multimode entangled states. We prepare Bell states [19] between two modes with the following protocol: starting with the transmon in its excited state, we swap half of the excitation via a sideband pulse ($\sqrt{\text{iSWAP}}$) to the first mode. This creates a $|\Psi^+\rangle$ Bell state between the first mode and the transmon:

$$|\Psi^+\rangle = \frac{1}{\sqrt{2}}\left(|g100...0\rangle + |e000...0\rangle\right). \tag{40}$$

We can rotate this state into the $|\Phi^+\rangle$ Bell state by flipping the transmon state via its charge bias control:

$$|\Phi^+\rangle = \frac{1}{\sqrt{2}}\left(|g000...0\rangle + |e100...0\rangle\right). \tag{41}$$

For either of the states in equations (40) and (41), we can create the corresponding Bell states between two arbitrary modes by simply swapping the transmon state to another mode.

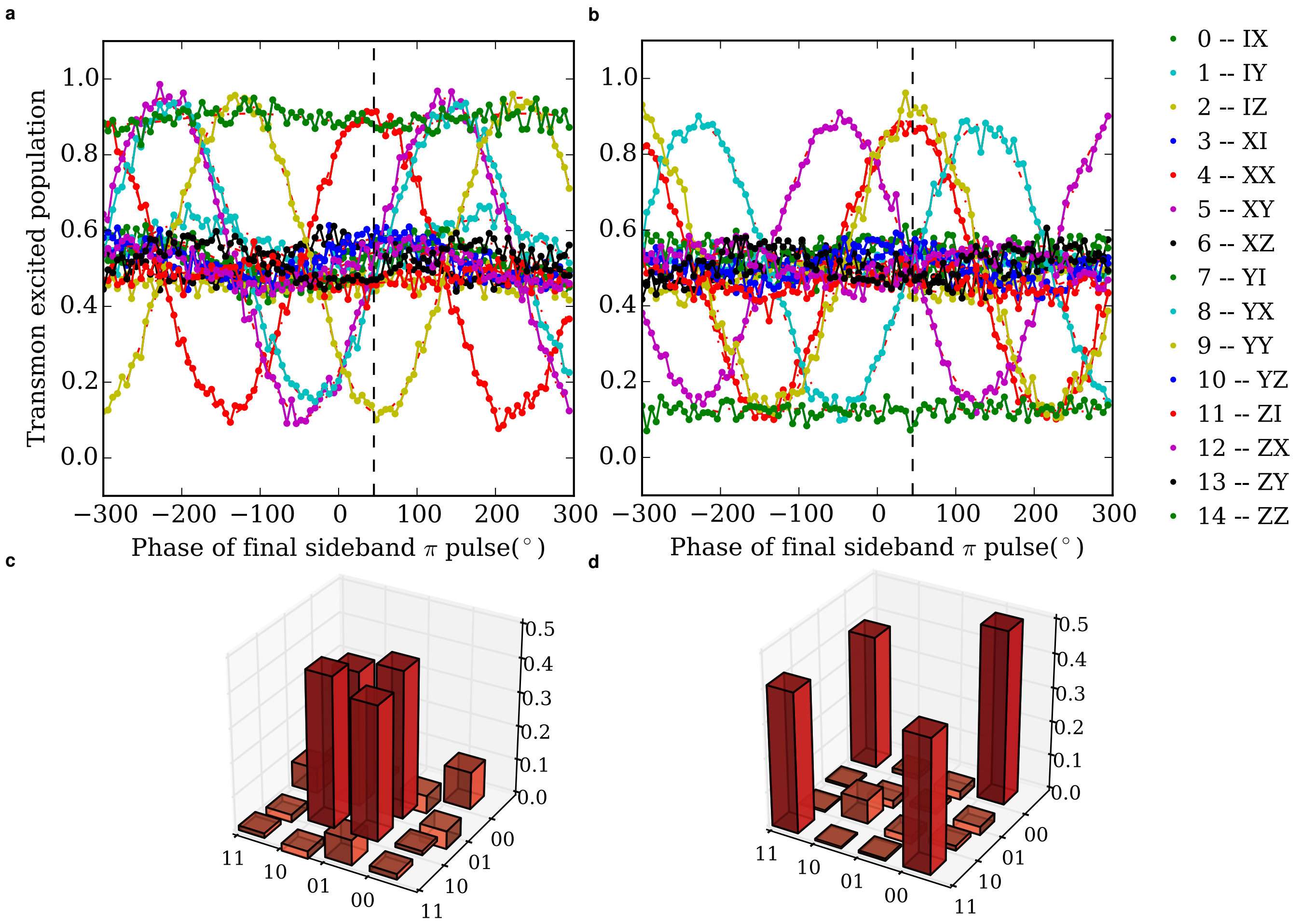

Supplementary Figure 20 | a and b, Transmon populations at the end of each of the two-mode correlator sequences for quantum state tomography of Ψ_+ and Φ_+ Bell states, respectively, as a function of the phase of the final sideband pulse. For these states, the only correlators that depend on the final sideband phase are $\mathrm{XX}, \mathrm{XY}, \mathrm{YX}, \mathrm{YY}$. Φ and Ψ Bell states are easily distinguished by a measurements of the two-mode parity operator ZZ. We account for additional phases by performing quantum state tomography using values measured at the sideband phase indicated by the dashed lines. **c and d,** The real parts of the density matrices. The corresponding state fidelities are found to be $F_p = 0.74$ and 0.75, respectively.

To extend this protocol and create a n-mode Greenberger-Horne-Zeilinger (GHZ) state [20], we again utilize the anharmonicity of the transmon and map the population of $|e\rangle$ to $|f\rangle$. This allows us to transfer this excitation to the second mode via a sideband of the $|e\rangle - |f\rangle$ transition without disturbing the population in the ground state:

$$|\psi\rangle = \frac{1}{\sqrt{2}} \left(|g000...0\rangle + |e110...0\rangle \right). \tag{42}$$

These last two pulses can be repeated for each of the remaining eigenmodes before finally swapping the transmon back to the nth mode to complete the GHZ state:

$$|\psi_{\mathrm{GHZ}}\rangle = \frac{1}{\sqrt{2}} \left(|g000...0\rangle + |g111...1\rangle \right). \tag{43}$$

Supplementary Note 19 – GHZ entanglement witness

We witness the entanglement of the 3-qubit GHZ states through a measurement of the Mermin operator [21, 22],

$$\mathcal{W} = \mathrm{XXX} - \mathrm{YYX} - \mathrm{YXY} - \mathrm{XYY}. \tag{44}$$

For an ideal n-qubit GHZ state, the Einstein-Podolsky-Rosen (EPR) [23] bound for $\langle \mathcal{W} \rangle$ is $2^{(n-1)/2}$, while quantum mechanics predicts $\langle \mathcal{W} \rangle = 2^{n-1}$. For the 3-qubit GHZ state, $\langle \mathcal{W} \rangle > 2$ demonstrates tripartite entanglement.

We measure 3-qubit correlators by including an additional two-mode gate to the pulse sequences in Table 4 for measuring 2-qubit correlations. The XXX correlator is measured through the experimental sequence shown in Supplementary Figure 21a. We reduce tomography error by compiling consecutive $|g\rangle - |e\rangle$ iSWAP pulses between successive CNOT gates, as well as the CNOT gate and the final iSWAP pulse. Additionally, we mitigate loss arising from transmon T_2^* by inserting a π pulse prior to the final $\pi/2$ qubit pulse, analogous to a spin-echo. The remaining correlators comprising $\mathcal{W}$ are measured by changing the phases of the CX gates and the final $\pi/2$ pulse. The results of the measurement of $\mathcal{W}$, as a function of the time τ between the π and $\pi/2$ pulses are shown in Supplementary Figure 21b. The echo results in a revival of $\langle \mathcal{W} \rangle$ at a time $\tau \sim 530\,\mathrm{ns}$. At the point indicated by the red star, $\langle \mathcal{W} \rangle = 2.35 \pm 0.04$, with the indicated error that arising from readout visibility (4×visibility error). While the echo mitigates errors from transmon dephasing, the measured value of $\langle \mathcal{W} \rangle$ is a lower bound that includes loss from tomography. The tomography sequence prior to the echo includes 5 sideband and 2 qubit π pulses. Accounting for the loss in these pulses based on the RB data for the $|g\rangle - |e\rangle$ iSWAPs and qubit pulses gives $\langle \mathcal{W} \rangle > 2.65$. This is still a conservative estimate for the witness, since the $|e\rangle - |f\rangle$ iSWAPs have a lower fidelity than the corresponding $|g\rangle - |e\rangle$ iSWAPs.

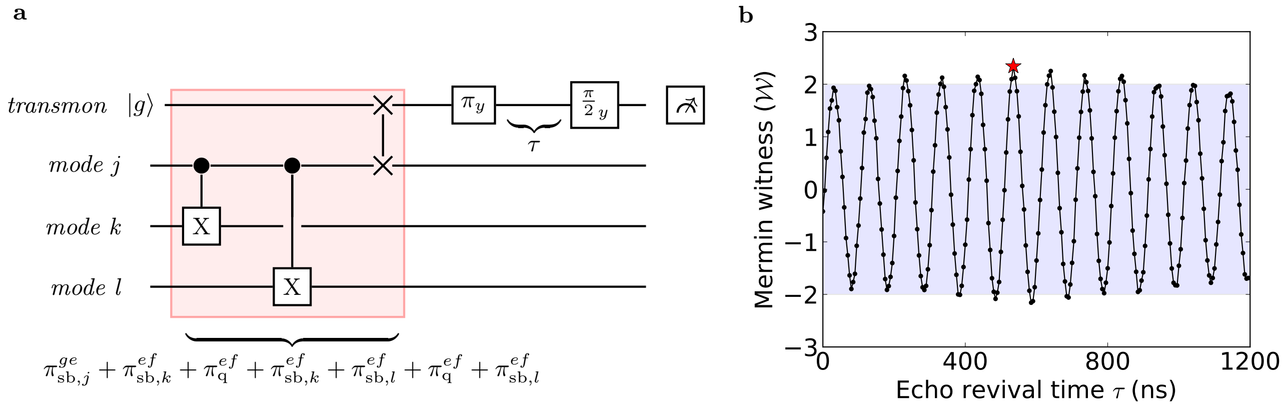

Supplementary Figure 21 | (a) Gate sequence for measurement of the XXX correlator with a transmon echo. As indicated by the pulse sequence, consecutive $|g\rangle - |e\rangle$ iSWAP pulses on the same mode in the red shaded region have been compiled and removed. (b) GHZ entanglement witness measured as a functon of time τ following the echo π pulse. The blue shaded region indicates the EPR bounds for $\mathcal{W}$, corresponding to the states being biseperable. The memory modes used in the measurement are $j = 6, k = 8$ and $l = 2$.

Supplementary References

[1] Wallraff, A. *et al.* Approaching unit visibility for control of a superconducting qubit with dispersive readout. *Phys. Rev. Lett.* **95**, 060501 (2005).

[2] Song, C., DeFeo, M. P., Yu, K. & Plourde, B. L. Reducing microwave loss in superconducting resonators due to trapped vortices. *App. Phys. Lett.* **95**, 232501 (2009).

[3] Gladchenko, S. *et al.* Superconducting nanocircuits for topologically protected qubits. *Nat. Phys.* **5**, 48–53 (2009).

[4] McKay, D. C., Naik, R., Reinhold, P., Bishop, L. S. & Schuster, D. I. High-contrast qubit interactions using multimode cavity QED. *Phys. Rev. Lett.* **114**, 080501 (2015).

[5] Beaudoin, F., da Silva, M. P., Dutton, Z. & Blais, A. First-order sidebands in circuit QED using qubit frequency modulation. *Phys. Rev. A* **86**, 022305 (2012).

[6] Strand, J. D. *et al.* First-order sideband transitions with flux-driven asymmetric transmon qubits. *Phys. Rev. B* **87**, 220505 (2013).

[7] Reed, M. D. *et al.* High-fidelity readout in circuit quantum electrodynamics using the Jaynes-Cummings nonlinearity. *Phys. Rev. Lett.* **105**, 173601 (2010).

[8] Bylander, J. *et al.* Noise spectroscopy through dynamical decoupling with a superconducting flux qubit. *Nat. Phys.* **7**, 565–570 (2011).
[9] Toll, J. S. Causality and the dispersion relation: logical foundations. *Phys. Rev.* **104**, 1760 (1956).
[10] Koch, J. *et al.* Charge-insensitive qubit design derived from the cooper pair box. *Phys. Rev. A* **76**, 042319 (2007).
[11] Ma, R., Owens, C., LaChapelle, A., Schuster, D. I. & Simon, J. Hamiltonian tomography of photonic lattices. *Phys. Rev. A* **95**, 062120 (2017).
[12] Knill, E. *et al.* Randomized benchmarking of quantum gates. *Phys. Rev. A* **77**, 012307 (2008).
[13] Chow, J. *et al.* Randomized benchmarking and process tomography for gate errors in a solid-state qubit. *Phys. Rev. Lett.* **102**, 90502 (2009).
[14] Ekert, A. K. *et al.* Direct estimations of linear and nonlinear functionals of a quantum state. *Phys. Rev. Lett.* **88**, 217901 (2002).
[15] Gottesman, D. The Heisenberg representation of quantum computers. *arXiv preprint quant-ph/9807006* (1998).
[16] Geerlings, K. *et al.* Demonstrating a driven reset protocol for a superconducting qubit. *Phys. Rev. Lett.* **110**, 120501 (2013).
[17] Haroche, S. & Raimond, J.-M. *Exploring the Quantum: Atoms, Cavities, and Photons* (Oxford University Press, 2006).
[18] O'Brien, J. L. *et al.* Quantum process tomography of a controlled-not gate. *Phys. Rev. Lett.* **93**, 080502 (2004).
[19] Bell, J. S. On the Einstein Podolsky Rosen paradox. *Physics* **1**, 195–200 (1964).
[20] Greenberger, D. M., Horne, M. A. & Zeilinger, A. Going beyond Bell's theorem. In *Bell's theorem, quantum theory and conceptions of the universe*, 69–72 (Springer, 1989).
[21] Mermin, N. D. Extreme quantum entanglement in a superposition of macroscopically distinct states. *Phys. Rev. Lett.* **65**, 1838 (1990).
[22] DiCarlo, L. *et al.* Preparation and measurement of three-qubit entanglement in a superconducting circuit. *Nature* **467**, 574–578 (2010).
[23] Einstein, A., Podolsky, B. & Rosen, N. Can quantum-mechanical description of physical reality be considered complete? *Physical Review* **47**, 777 (1935).
